# Enhancing palliative care in intensive care units: protocol of EPIC, a controlled, cluster-randomised, non-blinded stepped-wedge design trial with crossover phase

**DOI:** 10.1136/bmjopen-2025-108168

**Published:** 2026-02-17

**Authors:** Spyros D Mentzelopoulos, Christiane S Hartog, Theresa Tenge, Matthias Schwenkglenks, Sophie K Piper, Michaela Barbier, Katerina Rusinova, Martin Neukirchen, Stephen Schüürhuis, Hanne Irene Jensen, Vernon van Heerden, Jochen Dutzmann, Dominique Drescher, Markéta Zvara, Victoria Metaxa, Akiva Nachshon, Edoardo De Robertis, Claudia Spies, Andreas Edel

**Affiliations:** 1 National and Kapodistrian University of Athens Medical School, First Department of Intensive Care Medicine, Evaggelismos General Hospital, Athens, Greece; 2 Department of Anesthesiology and Intensive Care Medicine (CCM |CVK), Charité-Universitätsmedizin Berlin, Corporate Member of Freie Universität Berlin and Humboldt-Universität zu Berlin, Berlin, Germany; 3 Klinik Bavaria Kreischa, Kreischa, Germany; 4 tp21 GmbH, Berlin, Germany; 5 Interdisciplinary Center for Palliative Medicine, Medical Faculty, University Hospital Duesseldorf, Heinrich-Heine-University, Düsseldorf, Germany; 6 Department of Anesthesiology, Medical Faculty, University Hospital Duesseldorf, Heinrich-Heine-University, Düsseldorf, Germany; 7 Institute of Pharmaceutical Medicine (ECPM) and Health Economics Facility, Department of Public Health, University of Basel, Basel, Switzerland; 8 Institute of Biometry and Clinical Epidemiology, Charité-Universitätsmedizin Berlin, Corporate Member of Freie Universität Berlin and Humboldt-Universität zu Berlin, and Berlin Institute of Health, Berlin, Germany; 9 Institute of Medical Informatics, Charité-Universitätsmedizin Berlin, Corporate Member of Freie Universität Berlin and Humboldt-Universität zu Berlin, and Berlin Institute of Health, Berlin, Germany; 10 Department of Palliative Medicine, 1st Faculty of Medicine, Charles University, General University Hospital in Prague, Praha, Czech Republic; 11 Departments of Anesthesiology and Intensive Care, Lillebaelt Hospital, University Hospital of Southern Denmark; Department of Regional Health Research, University of Southern Denmark, Odense, Denmark; 12 Department of Anesthesiology, Critical Care and Pain Medicine, Faculty of Medicine, Hadassah Medical Center, Hebrew University of Jerusalem, Jerusalem, Israel; 13 Department of Internal Medicine III, Martin-Luther-University Halle-Wittenberg, University Hospital Halle (Saale), Halle, Germany; 14 Department of Critical Care, King’s College Hospital NHS Foundation Trust, London, UK; 15 Section of Anaesthesia, Analgesia and Intensive Care, Department of Medicine and Surgery, University of Perugia, Perugia, Italy

**Keywords:** INTENSIVE & CRITICAL CARE, PALLIATIVE CARE, Telemedicine, EDUCATION & TRAINING (see Medical Education & Training), HEALTH ECONOMICS, Person-Centered Care

## Abstract

**Introduction:**

Patients in intensive care units (ICUs) and their families face existential physical, psychosocial and spiritual distress. Integrating palliative care (PC) into ICU care may benefit patients, relatives and ICU clinicians. Prior PC studies have shown a reduction in ICU length of stay (LOS) and distressing symptoms without altering overall mortality. A shorter ICU LOS may alleviate the burden for patients and relatives and help optimise the use of limited intensive care resources. PC in the ICU, however, remains underused, partly due to limited access and knowledge of ICU clinicians. Also, robust data regarding the effectiveness and cost-effectiveness of PC treatment in the ICU are scarce. We established the ‘enhancing palliative care in ICUs’ (EPIC) study to implement a system-based harmonised practice model across European ICUs. The aim is to investigate if early integration of PC via telemedicine, clinician education and bedside tools is effective and cost-effective, ultimately benefiting patients, relatives and ICU clinicians.

**Methods and analysis:**

This multicentre, controlled, cluster-randomised, non-blinded stepped-wedge design trial with crossover phase aims to recruit around 2,000 patients from five European countries. All adult patients admitted to participating ICUs—with an ICU LOS exceeding 72 hours, where cancer is not the primary cause of critical illness, and who are not expected to die within the next 24 hours—are screened for the need for specialised PC based on the attending physician’s judgement. This judgement is triggered by the presence of one or more of the following: (1) significant disagreement among ICU team members and/or relatives about the appropriateness of current ICU treatment, (2) considerations of limiting life-sustaining therapy or (3) the anticipation that a specialised PC consultation may benefit the patient, their relatives or the ICU team. Patients identified as needing specialised PC and their relatives are then enrolled after obtaining written informed consent.

The complex intervention consists of (a) a blended-learning programme to foster knowledge and attitude about PC among ICU clinicians, (b) bedside tools, including a checklist to identify patients in need of PC and a factsheet and (c) standardised telemedical consultations from trained EPIC interventionists. Patient and relative follow-up is conducted 3 months post-ICU discharge. Outcomes include clinical measures (including ICU LOS (primary outcome), severity of critical illness, invasive treatments and health-related quality of life), economic endpoints (resource use, costs, cost–consequence situation, cost-effectiveness), ICU clinician burnout and distress, and patient and family perception about the quality of symptom management, care and communication. Endpoint analyses will employ generalised linear mixed models, accounting for the clustered data structure and stepped wedge design.

**Ethics and dissemination:**

EPIC complies with the Declaration of Helsinki and has been approved by all local ethics committees. A decision-making structure is established to ensure trial procedures are carried out according to Good Clinical Practice. Study findings will be published in peer-reviewed journals and communicated to participants, healthcare professionals and the public. Sets of anonymised study data will be made available following Findable, Accessible, Interoperable, and Reusable principles.

**Trial registration number:**

NCT06605079.

**STRENGTHS AND LIMITATIONS OF THIS STUDY**

- Multinational, multidisciplinary scope: the enhancing palliative care in intensive care units (EPIC) trial is the first European study conducted in multidisciplinary intensive care units (ICUs) across five countries to evaluate a palliative care (PC) intervention, assessing both clinical (such as ICU length of stay, symptom management and health-related quality of life) and economic endpoints, thereby contributing ground-breaking data in several dimensions.
- Interdisciplinary collaboration: conducted by a consortium of intensive care and PC experts, the pragmatic study fosters collaboration between distinct clinical cultures in real-world settings.
- Innovative educational and consultative approach: the intervention employs a consultative telemedicine PC model supported by a novel blended-learning programme designed for ICU nurses and physicians, thereby advancing both clinical practice and professional training.
- Pragmatic comparison with standard care: comparison with standard care across different countries and centres introduces a degree of heterogeneity, but also reflects the real-world complexity of ICU practice and enhances the generalisability of the findings.
- Patient enrolment affected by ‘subjectivity’: the inclusion criterion ‘need for specialised PC’ based on subjective assessment by the treating physician is partially subjective, reflecting how patients’ needs are assessed in real-world settings and offering an opportunity for clinical practice improvement through the intervention.

## Introduction

 Intensive care units (ICUs) are highly complex and demanding environments. Striking the right balance between technological advances and humane treatment can be challenging. The main focus of ICU care is to prolong a patient’s life, with death being perceived as treatment failure.^1^ ICU mortality varies according to age, comorbidity, critical illness diagnosis and treatment intensity and may reach 80% in specific vulnerable groups (eg, resuscitated cardiac arrest patients).^2^ Providing holistic ICU care without losing sight of the patient’s quality of life and well-being is crucial.

Palliative care (PC) is defined as ‘specialised medical care for people with a serious illness focused on providing relief from the symptoms and stress of the illness’, with the goal to improve the quality of life for both patients and their families.^3^ It can be delivered at two levels: primary and specialised PC. Primary PC, provided by all healthcare providers, mostly focuses on symptom management,^4^ whereas specialised PC is delivered by trained multiprofessional teams in complex clinical situations with an emphasis on holistic support—including symptom control, effective communication, shared treatment decisions and psychosocial patient/family support.^5^

PC specialists can be part of ICU teams in an integrative model but can also advise intensive care physicians in a consultative approach.^6^ This requires an understanding of what PC is in the context of intensive care, as well as when and why it can be useful.^57^,^9^

### Previous findings on PC in the ICU

Several studies have explored PC in the ICU from multiple angles. In addition to work on trigger criteria for identifying patients in need,^10^,^12^ other investigations have evaluated the impact of PC interventions on both patient outcomes and system-based metrics.^6 13^ The literature consistently documents that integrating PC into ICU workflows can reduce ICU length of stay (LOS) without altering overall mortality.^13 14^ A shorter ICU LOS may help optimise the use of expensive and scarce intensive care resources.^15^ However, robust health economic data regarding PC services in the ICU are still limited.^16^

Moreover, early and timely PC in the ICU can:

- Address the physical, psychosocial and spiritual suffering of patients and support families and loved ones.
- Help in prognostic assessment and the establishment of shared therapeutic goals.
- Enhance collaboration among intensivists, PC physicians, nurses, psychologists and social workers.^8^

Despite these promising findings, PC remains underused in the ICU setting.^17^

### Telemedicine PC

The integration of telemedicine into PC (tele-PC) is defined as the application of telemedicine technologies in the field of PC and presents a promising avenue for addressing the limited local availability of expertise.^18^ Telemedicine may offer an accessible, sustainable and potentially effective approach to deliver PC to ICUs, although evidence—primarily accumulated during the COVID-19 pandemic—remains sparse and randomised controlled trials are lacking.^18^,^20^ Nevertheless, tele-PC may support timely assessments and interventions.^21^

### Rationale of the research project

In light of the substantial burden of morbidity and mortality in the ICU—and the potential added benefits of PC integration via telemedicine—we have established the ‘Enhancing Palliative Care in ICUs’ (EPIC) clinical study. This stepped-wedge trial aims to demonstrate that early integration of PC supporting ICU clinicians by education, bedside tools and the provision of telepalliative care consultations can be achieved through a systems-based approach aligned with a harmonised European care model.

We anticipate that the EPIC study will benefit patients and healthcare staff by optimising care delivery and resource utilisation.

### Objectives and hypotheses

The study will address the following specific objectives:

- Clinical outcomes: assess the impact of the complex intervention on ICU LOS, the duration of mechanical ventilation and other organ replacement interventions and the proportion of patients with limitations of life-sustaining therapies.
- Quality of life: assess the impact of the intervention on health-related quality of life (HRQoL).
- Economic evaluation: estimate impact on resource use, analyse cost implications and determine the cost-effectiveness and cost–consequence situation of the intervention.
- Perception of care: evaluate whether patients and their relatives experience improved care quality and communication in the ICU, particularly in relation to end-of-life care and symptom management.
- Staff well-being: examine whether ICU clinicians (both physicians and nurses) report fewer stressors and perceive an improved ethical decision-making climate and practice.

The primary hypothesis is that the complex intervention will reduce the average ICU LOS by at least 2 days compared with standard care before. Secondary hypotheses include an increase in treatment limitation decisions; improved economic efficiency; enhanced perceived quality of care (notably in symptom management and shared decision-making); an improvement in HRQoL among survivors; and ameliorated moral distress among ICU clinicians.

## Methods

### Overview

**Figure 1:**
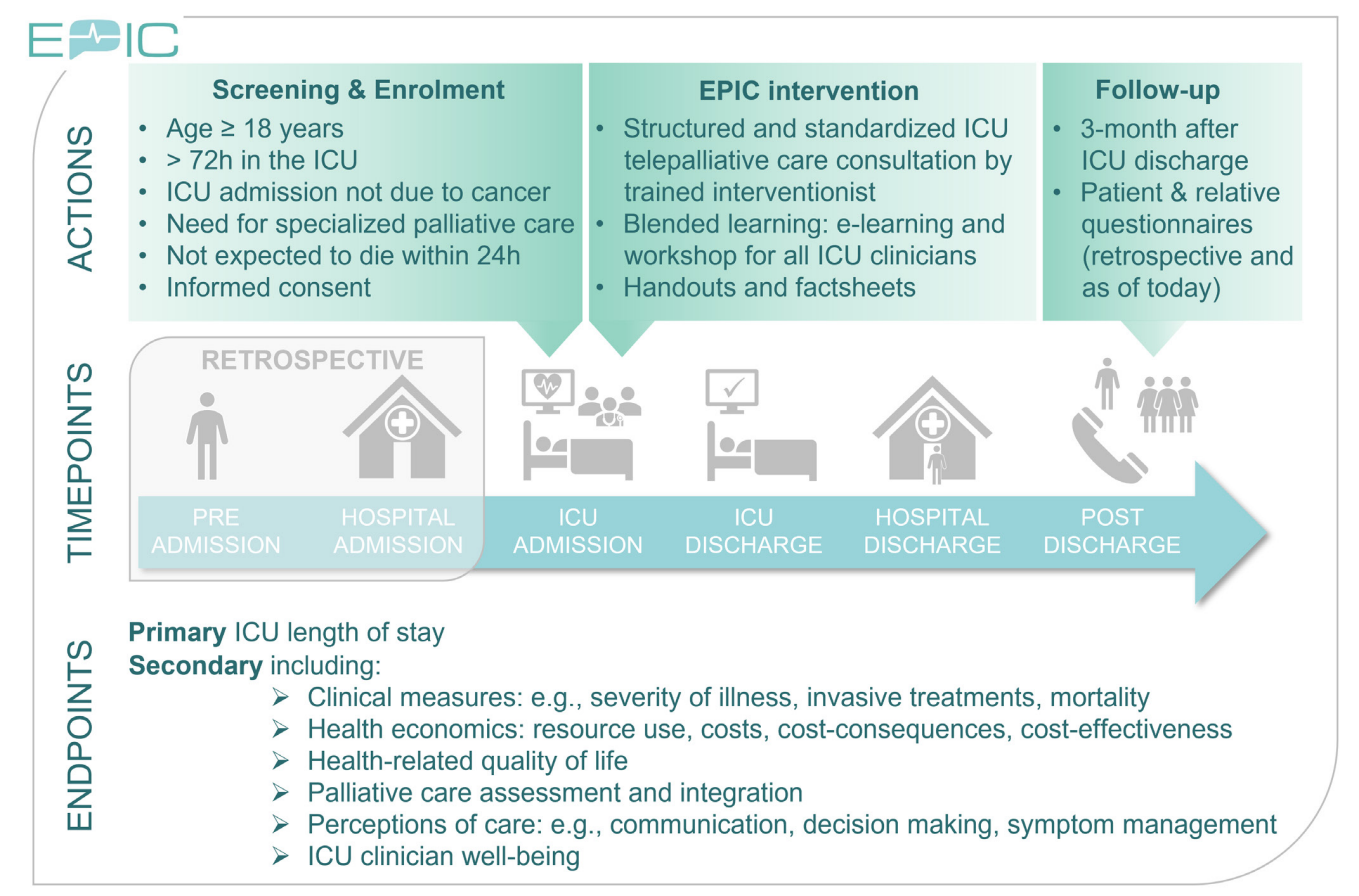
Study overview. Adult patients are screened for eligibility after intensive care unit (ICU) admission. Once enrolled, endpoints/study data are collected retrospectively (ie, for preceding time points as indicated in the grey box) and prospectively (ie, from the time point of enrolment). The enhancing palliative care in ICUs (EPIC) intervention is implemented within the ICU. At discharge, the ICU length of stay is recorded as the primary endpoint, with additional secondary endpoint data concurrently collected. Follow-up occurs at 3 months after ICU discharge and includes questionnaires for both patients and their relatives, along with retrospective collection for the time point of day 28 after ICU discharge.

We intend to complete a controlled, cluster-randomised, non-blinded stepped-wedge design trial with crossover phase to compare the intervention with standard treatment. Each cluster is formed by all participating ICUs sharing staff within one building or healthcare institution. Standard treatment is provided during the observation phase and the intervention is delivered during the subsequent intervention phase. All ICUs start with the observation phase. All patients who fulfil the eligibility criteria, including the ICU physician’s assessment of the need for specialised PC, are asked to participate in the study. At a randomised time point, each ICU cluster enters a crossover period of 1 month (see online supplemental material figure 1). Within this month, ICU staff receive online training in basic PC and EPIC interventionists are trained to conduct telepalliative consultations. In the subsequent intervention phase, the ICU physicians can use a newly developed checklist to assess specialised PC needs and schedule at least one specialised PC consultation via telemedicine. Survivors’ and relatives’ outcomes are gathered at pre-specified time points, that is, on ICU/hospital discharge and during a 3-month follow-up interview. Clinician outcomes (concerning burnout, end-of-life practices and ethical decision-making) are collected through a dedicated survey scheduled repeatedly in the control and intervention periods. The trial design is illustrated in figure 1.

This trial protocol is presented in accordance with the Standard Protocol Items for Interventional Trials^22^ and also takes into account the Consolidated Standards of Reporting Trials guideline for stepped-wedge cluster randomised trials,^23^ the Standards for Quality Improvement Reporting Excellence guideline^24^ and the Template for Intervention Description and Replication checklist.^25^ Additional details are provided in the online supplemental materials 2–5.

The project’s webpage (https://epic4icu.eu/) provides an overview for clinicians, patients and their relatives.

### Study setting

The study is currently being conducted in seven coordinating clinical centres and numerous multidisciplinary ICUs across five European countries (Czech Republic, Germany, Greece, Israel and Italy). Each coordinating clinical centre is designated as a ‘hub’ providing specialist teleconsultations to remote ICUs, which act as ‘satellites’ where patients are enrolled. The satellite ICUs encompass the full range of intensive care levels.

### Eligibility criteria

Identical enrolment criteria apply for both the observation and intervention phase; therefore, throughout the study period, all consecutive patients who are admitted to a participating ICU will be routinely screened for the following enrolment criteria.

#### Inclusion criteria

- ICU patients with decisional capacity, who can give informed consent for study participation, and incapacitated patients with an available, legally recognised, substitute decision-maker able to provide informed consent on their behalf.
- Adults aged 18 years and more.
- The leading cause of critical illness is not cancer; such patients represent a clinically distinct group with different prognosis, pre-existing PC involvement and oncology-specific care pathways that would likely confound the trial’s effect on ICU LOS and other outcomes.^26 27^ Moreover, the most significant evidence gap in the literature on PC for critically ill patients concerns non-cancer populations,^26^,^28^ providing a strong rationale for the inclusion criterion and the design of the present trial.
- New admission in the participating ICU, with LOS of >72 hours.
- Need for specialised PC according to attending physician judgement, based on (a) significant disagreement among ICU team members and/or family members about appropriateness of ICU treatment intensity or (b) consideration of therapy limitations or (c) expected benefit from specialised PC consultation for the patient/family and/or ICU team members.

#### Exclusion criteria

- Moribund patients expected to die within 24 hours.

### Study procedures

Each participating ICU cluster goes through the below-described consecutive phases.

### Observation phase: control condition

The care and treatment of the patient is carried out in the participating ICU according to local standards of care. If patients require specialised PC, and this is available, it is provided in person according to local practice.

### Crossover phase: clinician education, bedside tools and interventionist training

In the crossover month, the Interdisciplinary Center for Palliative Medicine of the University Hospital Duesseldorf, Germany, provides a structured training programme to the physicians and nurses in the respective satellite ICUs (programme description available at https://clinicaltrials.gov/study/NCT06425692?term=enhancing-palliative-care-&rank=2). Training in PC is carried out online in a blended learning format^29^ with an interactive e-learning course and interactive webinars.^30^ Training impact is assessed by comparative self-assessment^31^ to assess predefined learning gains, and by the continuing professional response questionnaire,^32^ a validated tool to identify behavioural change. Training participation rate is determined as the ratio of the actual number of participants to the total number of eligible staff members of the participating ICUs.

Additionally, a newly developed checklist of possible trigger factors (box 1) is provided to the ICU staff to raise awareness for PC needs of ICU patients and their families daily and guide the tele-PC consultations. The checklist is based on literature research and developed by the Delphi process among participating clinicians of the EPIC consortium. An additional bedside tool is a factsheet with recommended PC practices and relevant legislation developed for this study by the department of intensive care medicine of the National and Kapodistrian University of Athens, Greece.

Box 1 **Trigger checklist for palliative care needs for intensive care unit patients and their families**

- Frequent hospitalisations/emergency department visits
- Persistent/uncontrolled symptoms
- Explore patients’ values and preferences and align the care
- Assistance with ascertaining goals of care or end-of-life planning
- Patient/family request for palliative care involvement
- Existential distress or spiritual concerns

The EPIC interventionists (PC consultants) in each hub who provide the teleconsultation have received a variable range of PC education and therefore will receive structured training to ensure a standardised approach according to current standards^25^ (box 2). Training impact is assessed by pretraining and post-training evaluations, using feedback surveys. Intervention fidelity is assessed by periodic (quarterly) audits of consultations, adherence to the protocol and clinician self-reports as described in further detail below.

Box 2 **Key components of EPIC tele-interventionist training**

- Asynchronous training (part 1 and 2)—recorded PowerPoint presentations
- Synchronous training (booster sessions)
  - Monthly online meetings
  - Identifying areas of specific need and addressing them
  - Skill practice
  - Debriefing
  - Evaluation
- Content: part 1
  - The EPIC 6-item checklist
  - Understanding diagnosis, treatment and prognosis
  - Prognostic awareness (hopes and worries technique)
  - Empathetic statements
  - Delivering serious news
  - Conflict de-escalation techniques
- Content part 2
  - Distressing symptoms
  - Appropriateness of care concept
  - Goals of care framework
    - Reframe and focusing on current reality
    - Mapping values and priorities
    - Aligning care with patient preferences
    - Planning future care/discharge
  - Specific situations
    - Spiritual injury and the ‘miracle talk’

EPIC, Enhancing Palliative Care in the Intensive Care Unit.

PC trigger checklist and PC consultants’ training are part of the study contribution of the department of palliative medicine at the General University Hospital of Prague, Czech Republic.

### Intervention phase: telemedicine consultation by EPIC interventionists

The telemedicine framework enables secure, interactive, two-way audio-visual communication at the patient bedside, facilitating direct dialogue between PC consultants and the local ICU team, including attending physicians. For each patient, a minimum of one PC teleconsultation is conducted, using either commercial software with telerobots (‘high-tech’, Teladoc Health, Purchase, New York, USA) or standard videocall platforms (‘low-tech’,eg, Zoom Video Communications. Zoom. V.5.6.1, 2021, (https://zoom.us, used alongside McAfee Total Protection 2025). These methods, established during the COVID-19 pandemic, are practised according to local standards and regulatory requirements.

Teleconsultations are conducted with the attending physician and focus on PC assessments, including:

- Physical symptoms (eg, pain, dyspnoea, delirium, vomiting).
- Psychological distress for the patient and family (eg, anxiety, fear, lack of understanding of prognosis), as well as spiritual needs.
- Social issues affecting the patient and family (eg, childcare, financial challenges, discharge planning, social counselling).
- End-of-life communication between the treating team and the patient/family (eg, eliciting values, goals and preferences, understanding prognosis and treatment options, making shared decisions about treatment limitations).

Following these assessments, interventionists offer tailored advice for patient- and family-centred interventions on a case-by-case basis. All evaluations and proposed PC interventions are systematically recorded in the electronic case report form (e-CRF) of the study. A further detailed description of the telepalliative care consultation content is provided in online supplemental material 6.

### Implementation support and fidelity monitoring

Measures to support implementation include:

- Identification of local champions (eg, prestigious senior physicians or clinical researchers): these key facilitators support smooth communication between PC specialists and ICU teams, ensuring compliance and engagement.
- Personal introductory meetings (organised by country-level lead investigators): during the 4-week crossover period, PC specialists meet with ICU clinicians, staff and local champions to build trust and promptly identify potential challenges in protocol implementation, thereby promoting protocol adherence.^33^
- Legal framework factsheets: developed by EPIC researchers, these concise materials outline national laws relevant to end-of-life care, providing ICU-specific infographics.
- Communication skills handouts: ICU staff receive practical guides, covering essential topics such as delivering bad news and shared decision-making.
- Ethical practice prompts: during consultations, teams receive structured reminders to integrate ethical screening into PC discussions.

To ensure intervention fidelity, a three-step plan is implemented:

#### Training and ongoing supervision

EPIC consultants receive the comprehensive training mentioned above and presented in box 2.

#### Monitoring and documentation

Biweekly ICU screening/enrolment reports (provided by investigators during country-level and/or trial steering committee conference calls) detail the total number of enrolled patients, while monthly updates ensure timely data sharing for streamlined oversight.

#### Feedback and support

Feedback loops reinforce protocol adherence, supported by the aforementioned status updates from national coordinators and their associates. Furthermore, at each PC consultation, specialists review screening status, ensuring questionable cases are addressed.

National coordinators continue fostering strong partnerships across all study levels. Identified challenges are resolved through direct communication with clinicians or targeted protocol adjustments. These developments are discussed in three-monthly conference calls of the General Assembly/Executive Board.

### Outcome measures

#### Primary outcome

The primary outcome is the ICU LOS in days, operationalised as the total number of consecutive and/or non-consecutive ICU days from the start to the end of an index hospitalisation at a study-participating hospital; any transfer to another hospital for treatment continuation signifies termination of the index hospitalisation, and therefore, any subsequent ICU admission cannot contribute to the measured ICU LOS. Regarding index hospitalisation, whenever ICU discharge is followed by ICU readmission on the same day, ICU LOS is determined by taking into account the aforementioned day only once (rather than two times).

#### Secondary outcomes

Secondary outcomes are collected up to 3 months after the last day of ICU stay (ie, the day of ICU discharge that is not followed by any ICU readmission during the index hospitalisation); they are listed in tables1^7^.

**Table 1:** Clinical outcome measures (hospital stay)

Outcome measure	Measure description	Time frame
Maximum SOFA Score	Maximum sepsis-related organ failure assessment score, including a derived delta SOFA (total maximum SOFA minus admission SOFA) as a descriptor of multiple organ dysfunction.	During ICU stay (average ~7–9 days)
Incidence of delirium	Occurrence of delirium measured by validated delirium scoring instruments.	During ICU stay (average ~7–9 days)
ICU complications	Occurrence of any complication impacting length of stay and mortality. Captured by the question: CPR, tracheostomy, major surgery due to complication or none.	During ICU stay (average ~7–9 days)
Presence and nature of treatment limitations	Documentation of treatment limitations (eg, restrictions on frequency, scope or duration of interventions).	During ICU stay (average ~7–9 days)
ICU mortality (yes/no)	Whether the patient died during the ICU stay.	During ICU stay (average ~7–9 days)
Readmissions to ICU	The frequency at which patients, after being transferred out of the ICU, are readmitted to the ICU.	During hospital stay (expected average ~14–16 days)
Length of hospital stay	Total number of days from hospital admission to hospital discharge.	During hospital stay (expected average ~14–16 days)
Discharge destination (ICU)	Recorded destination on ICU discharge. Options include: hospital unit, other ICU in the hospital, external ICU, skilled nursing facility, rehabilitation unit, palliative care unit, hospice, home or other.	During hospital stay (expected average ~14–16 days)
Discharge destination (hospital)	Recorded destination on hospital discharge. Options include: skilled nursing facility, rehabilitation unit, palliative care unit, hospice, home or other.	During hospital stay (expected average ~14–16 days)
Hospital mortality (yes/no)	Whether the patient died during the overall hospital stay.	During hospital stay (expected average ~14–16 days)
Days of mechanical ventilation	Total number of days the patient received invasive mechanical ventilation.	During ICU/hospital stay
Days of ECMO	Total number of days the patient was supported by ECMO.	During ICU stay
Days of RRT	Total number of days the patient received renal replacement therapy (eg, dialysis).	During ICU/hospital stay
Days of LRT	Total number of days the patient received liver support therapy.	During ICU stay
Days of VADs	Total number of days the patient was supported by ventricular assist devices (eg, LVAD, RVAD, Impella, IABP).	During ICU/hospital stay

Impella: a percutaneous left ventricular assist device.

CPR, cardiopulmonary resuscitation; ECMO, extracorporeal membrane oxygenation; IABP, intra-aortic balloon pump; ICU, intensive care unit; LRT, liver replacement therapy; LVAD, left ventricular assist device; RRT, renal replacement therapy; RVAD, right ventricular assist device; SOFA, Sequential Organ Failure Assessment; VAD, ventricular assist device.

**Table 2:** Process and patient-centred/family-centred outcome measures (hospital stay)

Outcome measure	Measure description	Time frame
Quality of life and health status	Quality-of-life score as measured by the EQ-5D-5L plus a 0–100 VAS rating of overall health status; scores correspond to day 14 before ICU admission, to day 1 of study enrolment and to day of ICU discharge	Up to ICU discharge
Palliative care assessment	Whether a palliative care assessment was performed (yes/no).	During ICU stay (average ~7–9 days)
Specialised palliative care expert consultation	Documentation of whether a specialised palliative care expert was consulted (yes/no), reflecting added multidisciplinary support for patient and family.	Until hospital discharge (expected average ~14–16 days)

* Retrospective assessment of quality of life.

EQ-5D-5L, European Quality of life-5 Dimensions 5-Level; ICU, intensive care unit; VAS, Visual Analogue Scale.

**Table 3:** Patient follow-up outcomes (3-month follow-up)

Outcome measure	Measure description	Time frame
Level of distress at present time point	Rating on a numeric scale from 0 (no distress) to 10 (extreme distress).	Up to 3 months after ICU discharge
Anxiety and depression	Rating of responses to the four questions of the Patient Health Questionnaire 4, according to a four-point scale, ranging from ‘not at all’ to ‘nearly every day’.	Up to 3 months after ICU discharge
Satisfaction with quality of symptom control in the ICU	Rating of management of pain, breathlessness and anxiety/fear/agitation according to a five-point Likert scale, ranging from ‘excellent’ to ‘poor’.*	Up to 3 months after ICU discharge
Adequacy of explanation of treatment by ICU clinicians	Rating according to a four-point Likert scale, ranging from ‘very well’ to ‘not at all’.*	Up to 3 months after ICU discharge
Effort by ICU clinicians to obtain information about the patient’s treatment goals	Rating according to a four-point Likert scale, ranging from ‘very well’ to ‘not at all’.*	Up to 3 months after ICU discharge
Alignment of intensity and duration of ICU therapy with patient’s wishes	Rating according to a three-point Likert scale (‘too much’,‘just right’,‘too little’).*	Up to 3 months after ICU discharge
Quality of life and health status	Quality of life score as measured by the EQ-5D-5L plus a 0–100 VAS rating of overall health status; scores correspond to day 28† and day 90 after ICU discharge	Up to 3 months after ICU discharge
Facilitators for high-quality palliative care	Open-ended response identifying key factors that promote high-quality palliative care.	Up to 3 months after ICU discharge
Barriers to high-quality palliative care	Open-ended response identifying factors that hinder high-quality palliative care.	Up to 3 months after ICU discharge

* For all Likert-scale items, responses were offered on the stated scale along with an additional ‘no answer/can’t say’ option.

† Retrospective assessment of quality of life.

EQ-5D-5L, European Quality of life-5 Dimensions 5-Level; ICU, intensive care unit; VAS, Visual Analogue Scale.

**Table 4:** Relative follow-up outcomes (3-month follow-up)

Outcome measure	Measure description	Time frame
Satisfaction with quality of symptom control of loved one in the ICU	Rating of management of pain, breathlessness and anxiety/fear/agitation according to a five-point Likert scale, ranging from ‘excellent’ to ‘poor’.*	Up to 3 months after ICU discharge
Relative’s feeling of being included in ICU decision-making	Rating according to a five-point Likert scale, ranging from ‘very excluded’ to ‘very included’.*	Up to 3 months after ICU discharge
Adequacy of support for relative during ICU decision-making	Rating according to a five-point Likert scale, ranging from ‘totally unsupported’ to ‘very supported’.*	Up to 3 months after ICU discharge
Relative’s perceived control over patient’s ICU care	Rating according to a five-point Likert scale, ranging from ‘really out of control’ to ‘good control’.*	Up to 3 months after ICU discharge
Adequacy of time for the addressing of concerns and questions associated with decisions made during ICU stay	Rating according to a five-point Likert scale, ranging from ‘definitely inadequate’ to ‘substantial amount’.*	Up to 3 months after ICU discharge
Adequacy of explanation of loved one’s treatment by ICU clinicians	Rating according to a four-point Likert scale, ranging from ‘very well’ to ‘not at all’.*	Up to 3 months after ICU discharge
Effort by ICU clinicians to obtain information about the patient’s treatment goals	Rating according to a four-point Likert scale, ranging from ‘very well’ to ‘not at all’.*	Up to 3 months after ICU discharge
Perception of unnecessary prolongation of loved one’s life in the ICU	Rating according to a five-point Likert scale regarding the degree to which life was prolonged unnecessarily.*	Up to 3 months after ICU discharge
Evaluation of loved one’s comfort in their final hours before death in the ICU	Rating according to a five-point Likert scale, ranging from ‘very uncomfortable’ to ‘totally comfortable’.*	Up to 3 months after ICU discharge
Perception of healthcare team support for the relative during the loved one’s final hours before ICU death	Rating according to a five-point Likert scale, ranging from ‘very abandoned’ to ‘very supported’.*	Up to 3 months after ICU discharge
Facilitators for high-quality palliative care	Open-ended response on factors promoting high-quality palliative care, as perceived by relatives.	Up to 3 months after ICU discharge
Barriers for high-quality palliative care	Open-ended response on factors hindering high-quality palliative care, as perceived by relatives.	Up to 3 months after ICU discharge

* For all Likert-scale items, responses were offered on the stated scale along with an additional ‘no answer/can’t say’ option.

ICU, intensive care unit.

**Table 5:** Resource utilisation outcomes

Outcome measure	Measure description	Time frame
Days of mechanical ventilation	Total number of days the patient received invasive mechanical ventilation. (Note: mechanical ventilation may be provided both in ICU and non-ICU settings.)	During ICU/hospital stay
Days of RRT	Total number of days the patient received renal replacement therapy (eg, dialysis). (Note: RRT may be provided in both ICU and non-ICU settings.)	During ICU/hospital stay
Days of VADs	Total number of days the patient was supported by ventricular assist devices (eg, LVAD, RVAD, Impella, IABP). (Note: VAD support can occur both in and outside the ICU.)	During ICU/hospital stay
Days of ECMO	Total number of days the patient was supported by extracorporeal membrane oxygenation. (ECMO is exclusively provided in the ICU.)	During ICU stay
Days of LRT	Total number of days the patient received liver replacement therapy. (LRT is generally confined to the ICU.)	During ICU stay
Length of hospital stay	Total number of ICU and non-ICU days during index hospitalisation.	During hospital stay (expected average 14–16 days)
Outpatient resource utilisation	Number of outpatient visits the patient after the index hospitalisation, by type of provider.	Up to 3 months after hospital discharge
Rehospitalisations	Number and/or total days of rehospitalisations experienced after the index hospitalisation.	Up to 3 months after hospital discharge
Days in care institutions	Total number of days spent in rehabilitation centres, long-term care facilities or other care institutions after the index hospitalisation.	Up to 3 months after hospital discharge
Outpatient nursing services	Total amount of outpatient nursing care received (eg, number and duration of home nursing visits) after the index hospitalisation.	Up to 3 months after hospital discharge
Specialised outpatient palliative care	Use of specialised outpatient palliative care services (eg, number of visits) after the index hospitalisation.	Up to 3 months after hospital discharge
Provision of informal care by family members	Yes, or no, or unknown; if yes, number of hours per week	Up to 3 months after hospital discharge

Impella: a percutaneous left ventricular assist device.

ECMO, extracorporeal membrane oxygenation; IABP, intra-aortic balloon pump; ICU, intensive care unit; LRT, liver replacement therapy; LVAD, left ventricular assist device; RRT, renal replacement therapy; RVAD, right ventricular assist device; VAD, ventricular assist device.

**Table 6:** Health economics outcomes

Outcome measure	Measure description	Time frame
Costs	Comprehensive costs derived from hospital cost and billing data, including data on medical resource consumption and intervention costs (eg, staff time for palliative care activities combined with salaries per occupational group).	Measured during the index hospitalisation (ICU/hospital stay) and up to 3 months after hospital discharge
Cost-effectiveness, cost–consequence situation	Economic evaluation putting cost data in relation to patient and family outcomes.	Up to 3 months after hospital discharge

ICU, intensive care unit.

**Table 7:** Clinician-related outcome measures*

Outcome measure	Measure description	Time frame
Burnout assessment	BAT-12 Short version, 12 questions	Up to 34 months
Inappropriate therapy	Perception of inappropriate therapy, 5 questions, answer format 5-point Likert scale (ranging from ‘never’ agree to ‘very often’)	Up to 34 months
Moral distress	Moral distress is measured per employee using a (0–10) numeric rating scale	Up to 34 months
End-of-life practice	12 questions regarding the current implementation of end-of-life practice, yes–no answer format	Up to 34 months
Ethical decision-making climate in the ICU	8 items, answer format 5-point Likert scale (ranging from ‘strongly agree’ to ‘strongly disagree’)	Up to 34 months
Existence of standard operating procedures	4 questions about the presence of written ICU protocols, yes–no answer format	Up to 34 months
Use of the ABCDEF bundle	11 questions, yes–no answer format	Up to 34 months
Supporting measures for patient/family	4 relevant questions, yes–no answer format	Up to 34 months
Perception of palliative care and law	4 relevant questions, yes–no–don’t know answer format	Up to 34 months
Barriers to palliative care	A multiple choice question	Up to 34 months
Facilitators for palliative care	A multiple choice question	Up to 34 months
Facilitators and barriers for provision of palliative and end-of-life care through telemedicine consultations in the ICU	Three open-ended questions	Up to 34 months

* Clinician outcome data are collected via an electronic questionnaire repeatedly throughout the observation and intervention periods.

A, assess, prevent and Manage pain—ensuring effective pain management using validated scoring instruments; B, Both Spontaneous Awakening Trials and Spontaneous Breathing Trials—performing daily trials to reduce sedation and evaluate readiness for weaning from mechanical ventilation; BAT, Burnout Assessment Tool; C, choice of analgesia and sedation—optimising the selection and management of sedative and analgesic medications; D, delirium—assess, prevent and manage—routinely monitoring for delirium using validated tools and applying preventive/treatment strategies; E, early mobility and exercise—promoting early physical activity and rehabilitation to minimize ICU-acquired weakness; F, family engagement and empowerment—involving family members in care decision; ICU, intensive care unit.

#### Participant timeline

The data collection timeline is represented in table 8. The collection of secondary outcomes throughout follow-up is also performed for patients who have died before its completion time point (ie, at 3 months after ICU discharge), as applicable.

**Table 8:** Summary presentation of data collection schedule

	Visit 1 study inclusion	Visit - teleconsultation	Visit 2 ICU discharge*	Visit 3 follow-up 3 months post-ICU discharge	
Control phase (CP) Intervention phase (IP)	CP/IP	IP	CP/IP	CP/IP	
Time (hours; days (d))	Any time after 72 hours	<48 hours after inclusion		28 d after ICU discharge (retrospective)	90 d after ICU discharge
Review of inclusion and exclusion criteria, medical history	x				
Information/declaration of consent by legally recognised, substitute decision-maker	x				
Information/declaration of consent by patient	x				
Primary endpoint (ICU length of stay)	x		x		
Secondary endpoints	x	x	x	x	x
Mortality	x	x	x	x	x

* Defined as the last day of ICU stay (ie, the day of occurrence of ICU discharge that is not followed by any ICU readmission during the index hospitalisation); in case of more than one ICU admission during the index hospitalisation, the data on secondary endpoints are (repeatedly) collected on the corresponding days of ICU discharge; however, only the data collected on the last one of the aforementioned ICU discharge days are included in the secondary endpoints’ analyses.

ICU, intensive care unit.

### Sample size calculation

Primary endpoint is ICU LOS in days. We originally planned to recruit 23 ICUs for the trial, each recruiting two to three patients on average per month. Over the trial period of 35 months (including a crossover month without recruitment), we expected each ICU to recruit at least n=87 patients, resulting in at least n=23×87=2001 patients throughout the trial period. Assuming an average ICU LOS of 9 days during the control phase, a sample size of n=2001 patients in the trial would have yielded a power of approximately 85% at a two-sided significance level of 5% to detect a difference in ICU LOS between the intervention and control group of −2 days (SD±9 days).

This calculation was based on simulations using an intracluster correlation coefficient of 0.01 and various possible recruitment schemes with 2–3 average patients recruited per month per ICU. Reducing ICU LOS by 2 days means a clinically relevant reduction of burden by treatment and suffering of patients and their families. The calculations were performed with the software package R (V.4.1.2)^34^ and the programme package swCRTdesign (V.4.0).^35^

During the study initiation phase, 28 ICUs had confirmed participation. To mitigate potential cross-talk between ICUs located within the same institution, we grouped those sharing the same postal address into clusters. As a result, we randomised the crossover time point for each cluster (see online supplemental material figure 1A). Power calculations for this setup indicated an average statistical power of 85% for detecting the expected effect on ICU LOS.

Several months after initiation, two ICUs had dropped out because of delay in contract finalisation or non-recruitment. One ICU was replaced and additional in-house ICU units were added. In July 2025, it was decided to extend the control phase of clusters that had not yet transitioned into the intervention phase by 1 month, without extending the overall study duration of 35 months. This decision was made to mitigate recruitment delays during the control phase and reduce the associated design imbalance between the control and intervention groups. Based on the current recruitment rates and the initial planning assumptions, this modification would conservatively result in a power of approximately 80% at a two-sided significance level of 5% (see online supplemental material figure 1B for the adjusted study design chart).

### Patient recruitment and assignment of interventions

All consecutive patients and their next-of-kin fulfilling the eligibility criteria are invited to participate. The informed consent process is detailed below. Patient data are entered by on-site study personnel into the eCRF in secuTrial, which was set up by the Charité Clinical Trials Office.

Randomisation has been performed at the cluster level. Each cluster has been randomly assigned a time point for transitioning to the intervention phase during a crossover month, resulting in a permuted order of all clusters. The randomisation list was generated by the Institute of Biometry and Clinical Epidemiology of Charité-Universitätsmedizin Berlin using the R software package via random sampling without replacement.

### Data collection and management

#### Data collection and trial procedures during ICU stay and follow-up

Collected variables are summarised in figure 1 and tables1^8^; additional details are provided in the online supplemental material 7.

#### ICU treatment

Data documented by local research teams for all patients while in ICU include:

- Baseline data and critical illness characteristics (date and mode of hospital and ICU admission, ICU admission cause/diagnosis, eligibility criteria, documented pre-existing conditions/medical history, illness severity scores).
- Symptom burden (psychological, physical, social and spiritual) in the context of specialised PC consultation (intervention period only).
- HRQoL.
- ICU clinical course data (complications, organ replacement therapy, highest sequential organ dysfunction assessment score, delirium assessment/duration, treatment limitations).
- Hospital and ICU discharge data (discharge destination (intermediate care/normal care/death) and date).
- Patient details (unique identifiers, sociodemographic information, health insurance status).

#### Follow-up procedures and postacute care

Follow-up data are collected during a telephone interview with surviving patients and/or patients’ relatives, including HRQoL, use of health services and resources, satisfaction with care, symptom control, communication and decision-making, and perceived facilitators of and barriers to PC; the latter have been previously determined through research performed by the department of anesthesiology and intensive care, Lillebaelt University Hospital, Region of Southern Denmark. Survival status is determined at 3 months after ICU discharge from the patient, their relatives, general practitioner or municipal records/databases.

#### Medical resource use, cost and reimbursement data

Patient-level cost and billing data will be provided by the administrations of participating hospitals. Variables collected at the patient level will cover real costs of healthcare and reimbursements invoiced (including flat-fee reimbursement codes if applicable), between the start of the index hospitalisation and 3-month follow-up (see online supplemental material 8, figure 2).

Medical resource use data, including relevant admission and discharge dates, and information on informal care and resulting productivity losses are collected or extracted from clinical information systems by study staff (until the end of index hospitalisation) or self-reported by patients and relatives (from index hospitalisation’s end to the 3-month follow-up time point). This information will be combined with unit costs either stemming from publicly accessible, external sources or also provided by the hospital administrations. Centre-level unit costs requested from the hospital administrations will include, among others, average daily costs for an ICU, intermediate care or normal ward stay. Additionally, costs related to mechanical ventilation, renal or liver replacement and extracorporeal life support are collected, as well as different types of overhead expenses and salary levels (eg, ICU nurse, ICU physician, PC specialist).

The costs of the intervention will be estimated considering the cost of telemedicine technology (based on information from centres and/or technology providers), staff time (for training, PC consultations) and staff costs per unit of time, by relevant profession. The required information will be covered by the study data collection or provided anonymously/at the hospital level by the centres.

#### Data management

All clinical data collected during the study are stored in a bespoke secuTrial database (interactive Systems GmbH, Berlin, Germany) hosted by Charité-Universitätsmedizin Berlin and directly analysed from this database. Further details on data management, pseudonymisation and the data handling are provided in online supplemental material 9.

### Statistical methods

The statistical evaluation will be conducted by the Institute of Biometry and Clinical Epidemiology, Charité-Universitätsmedizin Berlin. Health economics analyses will be conducted by the Institute of Pharmaceutical Medicine and health economics facility, department of public health, University of Basel.

#### Data analysis plan

A detailed Statistical Analysis Plan will be finalised before database lock. The analysis of the data will begin with a detailed statistical description of the study sample, differentiated by control and intervention phase. Categorical characteristics will be summarised using absolute and relative frequencies. For ordinal or metric non-normally distributed data with up to five categories, we will present the absolute and relative frequencies, along with the median and the IQR. For data with more than five categories, only the median and IQR will be reported. For metric data that follows a normal distribution, we will present the mean value, accompanied by the SD and the 95% CI.

All analyses will be conducted on an intention-to-treat basis, with the patient representing the unit of analysis. We will use generalised linear mixed models (GLMMs) for continuous outcomes,^23 35^ accounting for the clustered data structure, to compare the primary endpoint between the control and intervention phases. Sensitivity analyses will include additional adjustment for ICU mortality.

Predefined subgroup analyses will be conducted to explore the heterogeneity of the treatment effect in patients concerning sex, religiosity, socioeconomic parameters and educational level. In addition, treatment effects will be compared between ICUs with high-tech versus low-tech telemedicine consultations, with good versus average or poor ethical climate, and ICUs with and without pre-existing palliative counselling. Further comparisons will be made among participating countries, patients with an ICU stay at enrolment of less than 5 days versus at least 5 days, and patients with versus without decisions to limit treatment at enrolment.

Subgroup analyses and analyses of all secondary endpoints will be considered as exploratory. Statistical testing will be carried out at a two-sided significance level of 5%. For the primary hypothesis, no correction for multiple testing is necessary.

#### Missing values

We anticipate that some patients or their relatives may withdraw from the study until the end of the hospital stay. We are accounting for this dropout during patient recruitment. Moreover, we will report frequency and possible reasons for any missing data. For secondary endpoints, we do expect missing data due to patient dropout in the follow-up telephone call and will report frequency and possible reasons accordingly. In the health economic analysis, missing values may be imputed based on the final dataset. The specific imputation method will be detailed separately in the Health Economic Analysis Plan (HEAP).

#### Health economic evaluation

Analytical details will be provided in the HEAP, which will also be finalised until project month 30. Health economic analyses involving costs are necessarily country specific. We will establish resource use and cost implications, and cost-effectiveness (economic efficiency), of the intervention from the healthcare system, societal and institutional (centre) perspectives, for Germany and at least one other country (ideally, all other countries depending on local interest and support with the identification of unit cost data). As the standard cost-effectiveness metric (costs per quality-adjusted life year (QALY) gained) is potentially challenging to interpret in PC situations, the cost results will also be related to other trial outcomes in a cost–consequence analysis. The costs of the intervention will be estimated and considered at the patient-level economic analyses on a pro rata basis.

Analyses will follow the principles applied for the primary endpoint and clinical analyses, to the extent they are applicable. Cost will be calculated by combining resource use and informal care data with suitable unit costs or will be directly provided by the administrations of participating centres. QALYs will be calculated from utilities (derived from European Quality of Life-5 Dimensions 5-Level (EQ-5D-5L) Questionnaire responses by applying published algorithms) using standard area under the curve methods following the trapezium rule.^36^ Costs, QALYs and additional outcome parameters that may be of interest for the cost–consequence analysis part will initially be described using standard methods. The presence of international variation/heterogeneity will be explored. Incremental costs and effects will then be assessed using suitable statistical models, typically GLMMs^23^ considering the specific requirements of the stepped-wedge design and the distributional characteristics of the studied parameters (eg, expected right-skewed distributions of resource use and cost parameters). Alternative analytical methods will be performed as robustness checks. For example, in order to capture the potential correlation of incremental costs and effects, the use of Generalized Structural Equation Models^37^ in the stepped-wedge setting will be explored. Depending on the amount of missing values, multiple imputations may be applied. We will perform rigorous uncertainty analyses combining non-parametric bootstrap-based estimation of uncertainty ranges and probabilistic sensitivity analysis of parameters affected by second order uncertainty (eg, unit costs).

### Ethics and dissemination

#### Research ethics approval

This study is conducted in accordance with ethical principles that have their origin in the Declaration of Helsinki and are consistent with Good Clinical Practice and EU General Data Protection Regulation. The protocol is based on the underlying project application to the European Union HORIZON Europe programme, which received previous independent peer review as part of the grant funding process. Together with the patient information sheets and consent forms, the protocol was first approved by the ethics committee of the Charité-Universitätsmedizin Berlin and subsequently by all participating centres’ ethics committees (table 9). The study is registered in ClinicalTrials.gov (NCT06605079).

**Table 9:** Ethics committees’ names and approval numbers

Country	Name of the ethics committee	Approval number
Germany	Charité’s Ethics Committee (Ethik-Kommission der Charité—Universitätsmedizin Berlin)	EA2/291/23
	Ethics committee at the medical faculty of Heinrich Heine University Düsseldorf (Ethikkommission an der Medizinischen Fakultät der Heinrich-Heine-Universität Düsseldorf)	2024-2823
	Ethics committee at Martin-Luther-Universität Halle-Wittenberg (Ethik-Kommission der Martin-Luther-Universität Halle-Wittenberg)	2024-063
Italy	Regional ethics committee of Umbria (Comitato Etico Regionale dell’Umbria)	4696/24
Czech Republic	Ethics committee of the General University Hospital in Prague (Etické komisi všeobecná fakultní nemocnice v Praze)	74/24 S Grant
Israel	Hadassah Medical Organization Institutional Review Board (ועדת הלסינקי להערכה מוסדית)	HMO-0062-24
Greece	National and Kapodistrian University of Athens Research Ethics Committee (Επιτροπή Ηθικής και Δεοντολογίας της Έρευνας (Ε.Η.Δ.Ε.) του Εθνικού και Καποδιστριακού Πανεπιστημίου Αθηνών)	125/2024
	Elena Venizelou—Alexandra General Hospital of Athens—Scientific Council (Γενικό Νοσοκομείο Αθηνών «Έλενα Βενιζέλου»—Αλεξάνδρα—Επιστημονικό Συμβούλιο)	99/22-02-2024
	Scientific council of the General University Hospital of Alexandroupolis (Πανεπιστημιακό Γενικό Νοσοκομείο Αλεξανδρούπολης—Επιστημονικό Συμβούλιο)	6851/05-02-2924
	General Hospital of Athens Evaggelismos-Polyclinic—Scientific Council—Ethics Committee («Γενικό νοσοκομείο Αθηνών Ευαγγελισμός—Πολυκλινική» Επιστημονικό Συμβούλιο—Επιτροπή Ηθικής και Δεοντολογίας)	320/19-06-2024
	Administrative Council of the General University Hospital of Ioannina (Διοικητικό Συμβούλιο του Πανεπιστημιακού Γενικού Νοσοκομείου Ιωαννίνων)	16/2-7-2024
	Administrative Council of the General University Hospital of Iraklion (Διοικητικό Συμβούλιο του Πανεπιστημιακού Γενικού Νοσοκομείου Ηρακλείου)	16/16-05-2024
	Scientific Council of the General University Hospital of Larisa (Επιστημονικό Συμβούλιο του Πανεπιστημιακού Γενικού Νοσοκομείου Λάρισας)	6/6/04-04-2024
	Scientific and ethics committee of the Onasis Cardiac Surgery Center (Επιστημονική Επιτροπή και Επιτροπή Ηθικής & Δεοντολογίας του Ωνασείου Καρδιοχειρουργικού Κέντρου)	806/13-05-2024
	Scientific Council of the General University Hospital of Patras ‘Holy Mary’s Help’ (Επιστημονικό Συμβούλιο του Πανεπιστημιακού Γενικού Νοσοκομείου Πατρών «Παναγία η Βοήθεια»)	10753/09-04-2024

#### Consent

Individual written informed consent including consent to data collection is obtained from all eligible patients, or their legal guardians, or their next-of-kin by the study personnel. The next-of-kin consent will be considered as valid, provided that it is explicitly recognised by local law.

#### Delayed consent procedure (‘deferred consent’)

If no legal guardian is available or has not been appointed or named at the time of the patient’s admission to ICU, and if there is no information in regards to the patient’s will (eg, living will or statements from relatives) to exclude participation in clinical research, the trial inclusion and thus the start of the trial (including training of doctors as part of the intervention) will only take place after the patient’s presumed consent (or next-of-kin consent) has been given. If the patient has generally ruled out participation in research, the trial inclusion and, thus, the start of the trial (eg, training of doctors as part of the intervention) only takes place after the patient’s presumed will with a positive attitude towards participation has been determined. If the patient is incapable of giving consent, the patient’s presumed will is determined by their environment or whether there is a conflicting will on the part of the patient. The patient’s presumed will regarding participation in the trial should be determined in a discussion with the relatives or a long-term partner. The patient’s presumed will is documented in a suitable form in the patient file and the trial folder with the involvement of relatives and/or future caregivers. The written consent of a confirmed legal guardian can be obtained for the patient’s participation. In the absence of a legal guardian, the patient’s next-of-kin consent will still be considered as valid, provided that this accords with local legislation. If the patient or their guardian, or their next of kin, refuses to participate in the trial or withdraws a previously given consent, the recorded data will be deleted (whenever explicitly requested).

#### Participant risks and study decision-making

By implementing a quality improvement intervention based on established procedures (teaching, checklist, teleconsultation), no additional risks to patients are expected relative to the accepted standard of care. A decision-making structure is established to ensure trial procedures are carried out according to Good Clinical Practice (see online supplemental material 10, figure 3).

#### Dissemination plan

Communication and dissemination of the EPIC project is described in a Dissemination, Exploitation and Communication Plan. Specifically, the results of the study will be published in peer-reviewed scientific journals and presented at national and international conferences. All publications will be regulated as described in the EPIC Publication Plan, with the support of a publication committee and based on communication rules set out by the European Commission. In addition, the study results will be communicated to patients, their representatives and healthcare professionals through press releases, professional societies and social media.

#### Patient and public involvement

Patient representatives have been involved in the initial planning of the study. A European Patient and Family Support Group is established by the University of Erlangen, Germany and will be involved in dissemination of the clinical trial results and in future practice recommendations.

#### Access to data

The metadata of all EPIC work packages will be made findable via registration in a repository (Zenodo). We create a Zenodo community where all EPIC outputs can be collected. Thus, every upload is assigned a digital object identifier, to make data citable and trackable.

Author, date created, date modified and file size are examples of very basic document metadata. Common European Research Information Format (https://eurocris.org/services/main-features-cerif) is the standard that the EU recommends to its member states for recording information about research activity.

The dataset, although findable, will not be publicly accessible, but a formal request for access to the data can be submitted to the ‘EPIC Data Access Committee’, specifically set up for this aim, which will define the ways of accessing data, the specific limitations, also regarding privacy requirements or limitations given by the consortium in a specific case.

## Discussion

The EPIC study is the first to investigate the impact of early integration of consultative PC in multidisciplinary European ICUs via telemedicine and an educational programme for ICU staff, implying a system-based harmonised practice model. The complex intervention fosters interdisciplinary collaboration and is assessed against standard care, with outcomes spanning patient clinical trajectories, family and clinician perceptions, and economic endpoints. Potential limitations include heterogeneity in ‘usual care’ across sites, subjectivity in trigger-based inclusion criteria, and the practical challenges of collecting patient-reported or proxy-reported data under high stress. In balancing feasibility with the high-quality analytic need to capture EQ-5D-5L data at five follow-up time points, we opted for two additional retrospective assessments as illustrated in figure 1. Intervention fidelity is monitored quarterly (every 3 months) via audits specified in our Implementation support and fidelity monitoring plan (see Methods), during which teleconsultation logs, trigger-checklist adherence and any relevant clinician comments (listed in the e-CRF) are reviewed.

Tele-PC consultations address a critical gap in ICUs by extending specialist expertise to units without onsite PC teams.^21^ Leveraging secure audio-visual platforms, EPIC enables timely symptom assessment, shared decision-making and goal clarification without in-person referral delays. During the COVID-19 pandemic, tele-PC maintained high clinician satisfaction and family engagement under visitor restrictions.^20^ By placing PC experts virtually at the bedside, EPIC overcomes geographic barriers and promotes consistent care delivery across diverse centres.^19^

Successful teleconsultation depends on structured implementation and robust training. EPIC’s blended-learning curriculum—interactive e-modules and live webinars—equips ICU teams with core palliative skills and the six-item trigger checklist for early need identification.^28^ Quarterly fidelity audits, empowered local ‘champions’ and clear standard operating procedures ensure adherence to EPIC’s protocol. This mirrors best practices from recent ICU palliative trials, which highlight the necessity of defined workflows, continuous feedback loops and sustained support to embed practice change.

Beyond operational feasibility, EPIC’s tele-PC model has the potential to enhance patient-centred and family-centred outcomes. Remote consultations expedite management of pain, delirium and psychological distress while fostering transparent conversations about prognosis and treatment preferences. Early qualitative feedback indicates improved family satisfaction with communication and involvement in care decisions, echoing findings that structured PC rounds—even via telemedicine—enhance surrogate engagement and reduce decisional conflict.^6^ Such patient-level benefits align with EPIC’s primary aim of shortening ICU LOS without adversely affecting survival. Furthermore, the EPIC study examines numerous secondary outcomes to strengthen the evidence base for early PC in the ICU, an area where current data are insufficient to support formal recommendations by the European Society of Intensive Care Medicine.^3^

The economic implications of tele-PC are noteworthy. By integrating specialist input remotely, EPIC may curtail unnecessary interventions and optimise resource use, translating into reduced ICU stays and cost savings. A systematic review found that ICU-based palliative consultations can shorten LOS by up to 2 days and yield net savings per patient episode.^15^ EPIC’s forthcoming cost–consequence analysis will refine these estimates across varied European health systems and inform scalable telehealth policy decisions.

Nonetheless, challenges persist. Variability in information technology infrastructure, data security regulations and digital literacy among staff can impede seamless teleconsultations, and heterogeneity of standard care across sites may modulate intervention effects and limit generalisability. Future research should explore hybrid models combining in-person and tele-PC, evaluate long-term outcomes beyond 3 months and investigate integration with emerging digital health platforms. Addressing these areas will be crucial to embed tele-PC as a routine component of critical care delivery across Europe.

## Supplementary material

10.1136/bmjopen-2025-108168 online supplemental file 1
