# Supplementary material for: Enhancing palliative care in intensive care units: protocol of EPIC, a controlled, cluster-randomised, non-blinded stepped-wedge design trial with crossover phase
Source: BMJ Open. 2026 Feb 17;16(2):e108168. doi: 10.1136/bmjopen-2025-108168 (PMC12918684; doi:10.1136/bmjopen-2025-108168)

BMJ Open – Protocol

Enhancing palliative care in intensive care units: protocol of EPIC, a controlled, cluster-randomized, non-blinded stepped-wedge design trial with crossover phase

SUPPLEMENTARY MATERIAL

Table of content

Supplementary material 1: Figure 1 – Stepped-wedge design 2
Supplementary material 2: Checklist 1 – Standard Protocol Items for Interventional Trials (SPIRIT) .. 4
Supplementary material 3: Checklist 2 – Standards for Quality Improvement Reporting Excellence (SQUIRE) guideline 9
Supplementary material 4: Checklist 3 – Consolidated Standards of Reporting Trials (CONSORT) guideline for stepped-wedge cluster randomized trials (SW-CRT) 11
Supplementary material 5: Checklist 4 – Template for intervention description and replication (TIDieR) checklist 14
Supplementary material 6: Methodological details 1 – Description of the telepalliative care consultation content 17
Supplementary material 7: References supporting the selection of the secondary outcomes 18
Supplementary material 8: Figure 2 – Detailed outcome overview 23
Supplementary material 9: Methodological details 2 – Data management, pseudonymization, data transfer and archiving 24
Supplementary material 10: Figure 3 – Decision making structure 26

Supplementary material 1: Figure 1 – Stepped-wedge design

A Original design

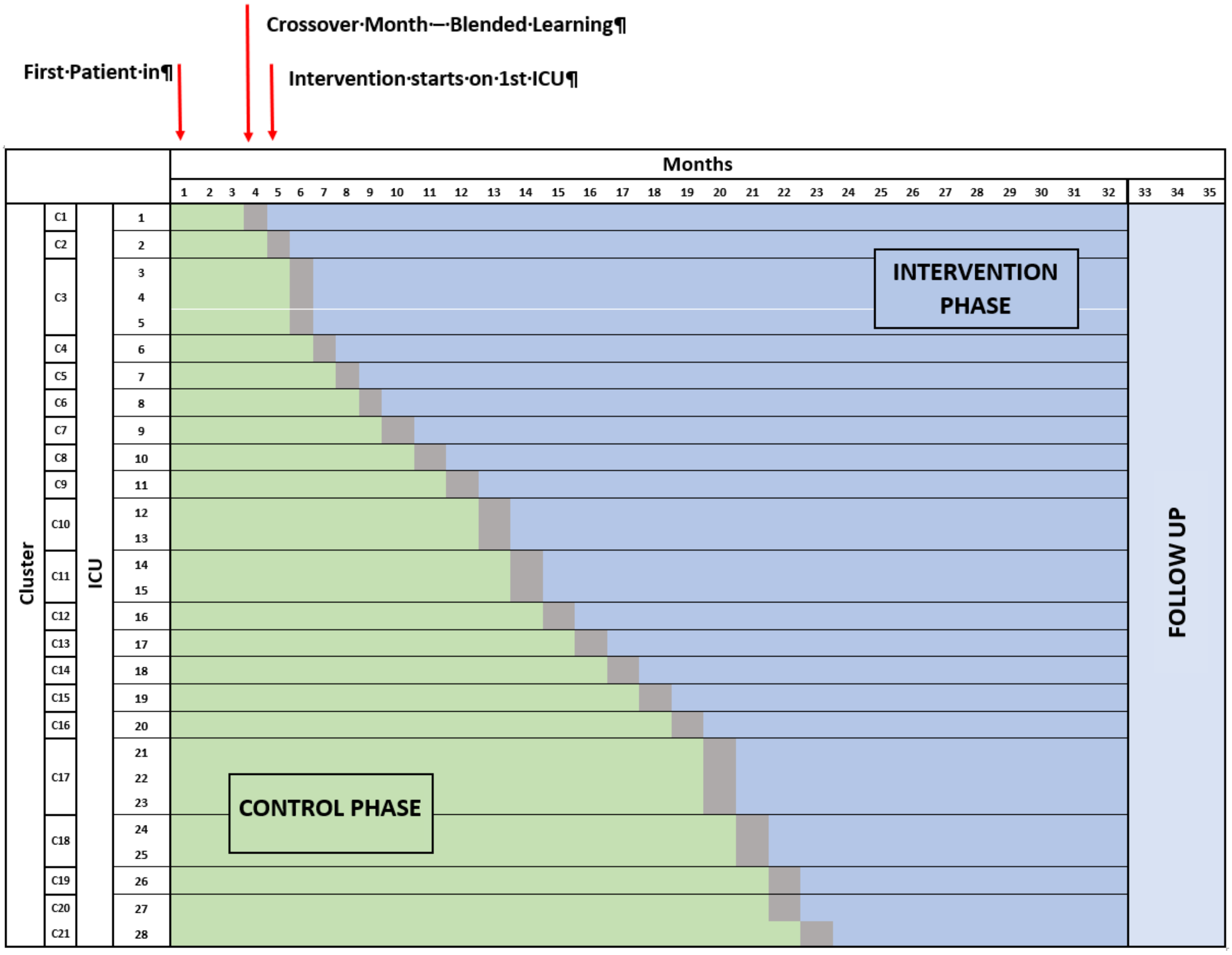

B Updated design with extension of the control phase by one month starting from cluster 7 (C7) in study month 10 (July 2025)

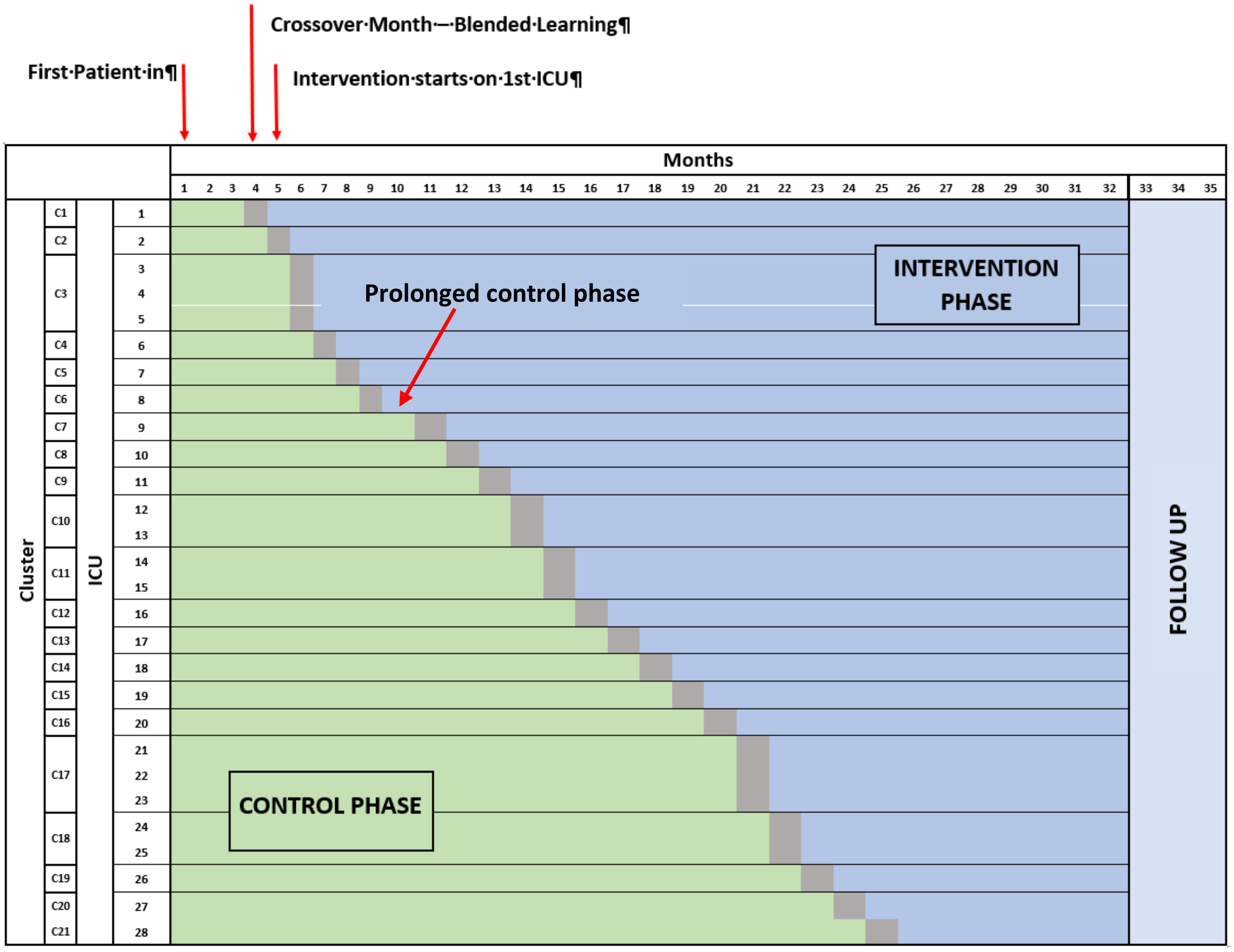

Supplementary material 2: Checklist 1 – Standard Protocol Items for Interventional Trials (SPIRIT)

**Section/item**	**Item No**	**Description**	**Addressed on page number**
**Administrative information**			
Title	1	Descriptive title identifying the study design, population, interventions, and, if applicable, trial acronym	1
Trial registration	2a	Trial identifier and registry name. If not yet registered, name of intended registry	2, 21
	2b	All items from the World Health Organization Trial Registration Data Set	N/A
Protocol version	3	Date and version identifier	2
Funding	4	Sources and types of financial, material, and other support	21
Roles and responsibilities	5a	Names, affiliations, and roles of protocol contributors	1
	5b	Name and contact information for the trial sponsor	21
	5c	Role of study sponsor and funders, if any, in study design; collection, management, analysis, and interpretation of data; writing of the report; and the decision to submit the report for publication, including whether they will have ultimate authority over any of these activities	21
	5d	Composition, roles, and responsibilities of the coordinating centre, steering committee, endpoint adjudication committee, data management team, and other individuals or groups overseeing the trial, if applicable (see Item 21a for data monitoring committee)	
**Introduction**			
Background and rationale	6a	Description of research question and justification for undertaking the trial, including summary of relevant studies (published and unpublished) examining benefits and harms for each intervention	5-7
	6b	Explanation for choice of comparators	7
Objectives	7	Specific objectives or hypotheses	6-7

Trial design	8	Description of trial design including type of trial (eg, parallel group, crossover, factorial, single group), allocation ratio, and framework (eg, superiority, equivalence, noninferiority, exploratory)	8
**Methods: Participants, interventions, and outcomes**			
Study setting	9	Description of study settings (eg, community clinic, academic hospital) and list of countries where data will be collected. Reference to where list of study sites can be obtained	9
Eligibility criteria	10	Inclusion and exclusion criteria for participants. If applicable, eligibility criteria for study centres and individuals who will perform the interventions (eg, surgeons, psychotherapists)	9-10
Interventions	11a	Interventions for each group with sufficient detail to allow replication, including how and when they will be administered	10-11
	11b	Criteria for discontinuing or modifying allocated interventions for a given trial participant (eg, drug dose change in response to harms, participant request, or improving/worsening disease)	17
	11c	Strategies to improve adherence to intervention protocols, and any procedures for monitoring adherence (eg, drug tablet return, laboratory tests)	11-12
	11d	Relevant concomitant care and interventions that are permitted or prohibited during the trial	9-11
Outcomes	12	Primary, secondary, and other outcomes, including the specific measurement variable (eg, systolic blood pressure), analysis metric (eg, change from baseline, final value, time to event), method of aggregation (eg, median, proportion), and time point for each outcome. Explanation of the clinical relevance of chosen efficacy and harm outcomes is strongly recommended	12-13, Tables 3-9
Participant timeline	13	Time schedule of enrolment, interventions (including any run-ins and washouts), assessments, and visits for participants. A schematic diagram is highly recommended (see Figure)	Figure 1,
Sample size	14	Estimated number of participants needed to achieve study objectives and how it was determined, including clinical and statistical assumptions supporting any sample size calculations	13-14
Recruitment	15	Strategies for achieving adequate participant enrolment to reach target sample size	14

**Methods: Assignment of interventions (for controlled trials)**			
Allocation:			
Sequence generation	16a	Method of generating the allocation sequence (eg, computer-generated random numbers), and list of any factors for stratification. To reduce predictability of a random sequence, details of any planned restriction (eg, blocking) should be provided in a separate document that is unavailable to those who enrol participants or assign interventions	13
Allocation concealment mechanism	16b	Mechanism of implementing the allocation sequence (eg, central telephone; sequentially numbered, opaque, sealed envelopes), describing any steps to conceal the sequence until interventions are assigned	9-18
Implementatio n	16c	Who will generate the allocation sequence, who will enrol participants, and who will assign participants to interventions	10-12
Blinding (masking)	17a	Who will be blinded after assignment to interventions (eg, trial participants, care providers, outcome assessors, data analysts), and how	N/A
	17b	If blinded, circumstances under which unblinding is permissible, and procedure for revealing a participant's allocated intervention during the trial	
**Methods: Data collection, management, and analysis**			
Data collection methods	18a	Plans for assessment and collection of outcome, baseline, and other trial data, including any related processes to promote data quality (eg, duplicate measurements, training of assessors) and a description of study instruments (eg, questionnaires, laboratory tests) along with their reliability and validity, if known. Reference to where data collection forms can be found, if not in the protocol	14-16
	18b	Plans to promote participant retention and complete follow-up, including list of any outcome data to be collected for participants who discontinue or deviate from intervention protocols	17
Data management	19	Plans for data entry, coding, security, and storage, including any related processes to promote data quality (eg, double data entry; range checks for data values). Reference to where details of data management procedures can be found, if not in the protocol	16, Suppl.

Statistical methods	20a	Statistical methods for analysing primary and secondary outcomes. Reference to where other details of the statistical analysis plan can be found, if not in the protocol	16-18
	20b	Methods for any additional analyses (eg, subgroup and adjusted analyses)	16-18
	20c	Definition of analysis population relating to protocol non-adherence (eg, as randomised analysis), and any statistical methods to handle missing data (eg, multiple imputation)	16-18
**Methods: Monitoring**			
Data monitoring	21a	Composition of data monitoring committee (DMC); summary of its role and reporting structure; statement of whether it is independent from the sponsor and competing interests; and reference to where further details about its charter can be found, if not in the protocol. Alternatively, an explanation of why a DMC is not needed	16, Suppl
	21b	Description of any interim analyses and stopping guidelines, including who will have access to these interim results and make the final decision to terminate the trial	16-18
Harms	22	Plans for collecting, assessing, reporting, and managing solicited and spontaneously reported adverse events and other unintended effects of trial interventions or trial conduct	N/A
Auditing	23	Frequency and procedures for auditing trial conduct, if any, and whether the process will be independent from investigators and the sponsor	11, 19
**Ethics and dissemination**			
Research ethics approval	24	Plans for seeking research ethics committee/institutional review board (REC/IRB) approval	21, Suppl.
Protocol amendments	25	Plans for communicating important protocol modifications (eg, changes to eligibility criteria, outcomes, analyses) to relevant parties (eg, investigators, REC/IRBs, trial participants, trial registries, journals, regulators)	14
Consent or assent	26a	Who will obtain informed consent or assent from potential trial participants or authorised surrogates, and how (see Item 32)	9

	26b	Additional consent provisions for collection and use of participant data and biological specimens in ancillary studies, if applicable	N/A
Confidentiality	27	How personal information about potential and enrolled participants will be collected, shared, and maintained in order to protect confidentiality before, during, and after the trial	16, Suppl.
Declaration of interests	28	Financial and other competing interests for principal investigators for the overall trial and each study site	N/A
Access to data	29	Statement of who will have access to the final trial dataset, and disclosure of contractual agreements that limit such access for investigators	21
Ancillary and post-trial care	30	Provisions, if any, for ancillary and post-trial care, and for compensation to those who suffer harm from trial participation	N/A
Dissemination policy	31a	Plans for investigators and sponsor to communicate trial results to participants, healthcare professionals, the public, and other relevant groups (eg, via publication, reporting in results databases, or other data sharing arrangements), including any publication restrictions	21, Suppl.
	31b	Authorship eligibility guidelines and any intended use of professional writers	1
	31c	Plans, if any, for granting public access to the full protocol, participant-level dataset, and statistical code	N/A
**Appendices**			
Informed consent materials	32	Model consent form and other related documentation given to participants and authorised surrogates	Suppl.
Biological specimens	33	Plans for collection, laboratory evaluation, and storage of biological specimens for genetic or molecular analysis in the current trial and for future use in ancillary studies, if applicable	N/A

*It is strongly recommended that this checklist be read in conjunction with the SPIRIT 2013 Explanation & Elaboration for important clarification on the items. Amendments to the protocol should be tracked and dated. The SPIRIT checklist is copyrighted by the SPIRIT Group under the Creative Commons "Attribution-NonCommercial-NoDerivs 3.0 Unported" license.

Supplementary material 3: Checklist 2 – Standards for Quality Improvement Reporting Excellence (SQUIRE) guideline

**Text section and item name**	**Page/line no(s). info is located**
**Title and abstract**	
1. **Title**	
Indicate that the manuscript concerns an initiative to improve healthcare (broadly defined to include the quality, safety, effectiveness, patient-centredness, timeliness, cost, efficiency and equity of healthcare).	1
2. **Abstract**	
a. Provide adequate information to aid in searching and indexing.	3-4
b. Summarise all key information from various sections of the text using the abstract format of the intended publication or a structured summary such as: background, local problem, methods, interventions, results, conclusions.	3-4
**Introduction: Why did you start?**	
3. **Problem description** - Nature and significance of the local problem.	5-6
4. **Available knowledge** - Summary of what is currently known about the problem, including relevant previous studies.	5-6
5. **Rationale** - Informal or formal frameworks, models, concepts and/or theories used to explain the problem, any reasons or assumptions that were used to develop the intervention(s) and reasons why the intervention(s) was expected to work	6
6. **Specific aims** - Purpose of the project and of this report.	6-7
**Methods: What did you do?**	
7. **Context** - Contextual elements considered important at the outset of introducing the intervention(s).	8
8. **Intervention(s)**	
a. Description of the intervention(s) in sufficient detail that others could reproduce it.	11-12
b. Specifics of the team involved in the work.	10-12
9. **Study of the intervention(s)**	
a. Approach chosen for assessing the impact of the intervention(s).	12-13
b. Approach used to establish whether the observed outcomes were due to the intervention(s).	12-18
10. **Measures**	
a. Measures chosen for studying processes and outcomes of the intervention(s), including rationale for choosing them, their operational definitions and their validity and reliability.	12-13
b. Description of the approach to the ongoing assessment of contextual elements that contributed to the success, failure, efficiency and cost.	12-15
c. Methods employed for assessing completeness and accuracy of data.	
11. **Analysis**	
a. Qualitative and quantitative methods used to draw inferences from the data.	16-18

b. Methods for understanding variation within the data, including the effects of time as a variable.	16-18
12. **Ethical considerations** - Ethical aspects of implementing and studying the intervention(s) and how they were addressed, including, but not limited to, formal ethics review and potential conflict(s) of interest.	21, Suppl.
**Results: What did you find?**	
13. **Results**	
a. Initial steps of the intervention(s) and their evolution over time (eg, time-line diagram, flow chart or table), including modifications made to the intervention during the project.	13-14
b. Details of the process measures and outcomes.	N/A
c. Contextual elements that interacted with the intervention(s).	13-14
d. Observed associations between outcomes, interventions and relevant contextual elements.	N/A
e. Unintended consequences such as unexpected benefits, problems, failures or costs associated with the intervention(s).	13-14
f. Details about missing data.	17
**Discussion: What does it mean?**	
14. **Summary**	
a. Key findings, including relevance to the rationale and specific aims.	N/A
b. Particular strengths of the project.	4, 19-20
15. **Interpretation**	
a. Nature of the association between the intervention(s) and the outcomes.	4, 19-20
b. Comparison of results with findings from other publications.	N/A
c. Impact of the project on people and systems.	4, 19-20
d. Reasons for any differences between observed and anticipated outcomes, including the influence of context.	N/A
e. Costs and strategic trade-offs, including opportunity costs.	N/A
16. **Limitations**	
a. Limits to the generalisability of the work.	4, 19-20
b. Factors that might have limited internal validity such as confounding, bias or imprecision in the design, methods, measurement or analysis.	4, 19-20
c. Efforts made to minimise and adjust for limitations.	4, 19-20
**Conclusions**	
a. Usefulness of the work.	19-20
b. Sustainability.	6
c. Potential for spread to other contexts.	19-20
d. Implications for practice and for further study in the field.	19-20
e. Suggested next steps.	20
**Other information**	
18. **Funding** - Sources of funding that supported this work. Role, if any, of the funding organisation in the design, implementation, interpretation and reporting.	21

Supplementary material 4: Checklist 3 – Consolidated Standards of Reporting Trials (CONSORT) guideline for stepped-wedge cluster randomized trials (SW-CRT)

Topic	Item no	Checklist item	Page no
**Title and abstract**			
	1a	Identification as a stepped-wedge cluster randomised trial in the title.	1
	1b	Structured summary of trial design, methods, results, and conclusions.<br>(See separate SW-CRT checklist for abstracts).	3-4
**Introduction**			
Background and objectives	2a	Scientific background. Rationale for using a cluster design and rationale for using a stepped-wedge design	5-6
	2b	Specific objectives or hypotheses	6-7
**Methods**			
Trial design	3a	Description and diagram of trial design including definition of cluster, number of sequences, number of clusters randomised to each sequence, number of periods, duration of time between each step, and whether the participants assessed in different periods are the same people, different people, or a mixture.	8-9
	3b	Important changes to methods after trial commencement (such as eligibility criteria), with reasons.	N/A
Participants	4a	Eligibility criteria for clusters and participants.	9-10
	4b	Settings and locations where the data were collected.	10-11
Interventions	5	The intervention and control conditions with sufficient details to allow replication, including how and when they were administered; whether the intervention was delivered at the level of the cluster, the individual, or both.	10-12
Outcomes	6a	Completely defined pre-specified primary and secondary outcome measures, including how and when they were assessed.	12-13
	6b	Any changes to trial outcomes after the trial commenced, with reasons	N/A
Sample size	7a	How sample size was determined. Method of calculation and relevant parameters with sufficient detail so the calculation can be replicated. Assumptions made about correlations between outcomes of participants from the same cluster.	13-14
	7b	When applicable, explanation of any interim analyses and stopping guidelines.	N/A
**Randomization**			
Sequence generation	8a	Method used to generate the random allocation to the sequences of treatments.	14
	8b	Type of randomisation; details of any constrained randomisation or stratification if used.	14
Allocation concealment	9	Specification that allocation was based on clusters; description of any methods used to conceal the allocation from the clusters until after recruitment.	14

mechanism			
Implementation	10a	Who generated the randomisation schedule, who enrolled clusters, and who assigned clusters to sequences	14
	10b	Mechanism by which individual participants were included in clusters for the purposes of the trial (such as complete enumeration, random sampling; continuous recruitment/ascertainment, or recruitment at a fixed point in time), including who recruited or identified participants.	14
	10c	Whether, from whom and when consent was sought and for what; whether this differed between treatment conditions.	14
Blinding	11a	If done, who was blinded after assignment to sequences (for example, cluster level participants, individual level participants, those assessing outcomes) and how.	N/A
	11b	If relevant, description of the similarity of treatments.	N/A
Statistical methods	12a	Statistical methods used to compare treatment conditions for primary and secondary outcomes including how time effects, clustering and repeated measures were taken into account.	16
	12b	Methods for additional analyses, such as subgroup analyses and adjusted analyses.	16-18
Participant flow (a diagram is strongly recommended)	13a	For each treatment condition or allocated sequence the numbers of clusters and participants who were assessed for eligibility, were randomly assigned, received intended treatments and were analysed for the primary outcome.	N/A
	13b	For each treatment condition or allocated sequence, losses and exclusions for both clusters and participants with reasons.	N/A
Recruitment	14a	Dates defining the steps, initiation of intervention and deviations from planned dates. Dates defining recruitment and follow-up for participants.	N/A
	14b	Why the trial ended or was stopped	N/A
Baseline data	15	Baseline characteristics for the individual and cluster levels as applicable for each treatment condition or allocated sequence.	N/A
Numbers analysed	16	The number of observations and clusters included in each analysis for each treatment condition and whether the analysis was according to the allocated schedule.	
Outcomes and estimation	17a	For each primary and secondary outcome, results for each treatment condition, and the estimated effect size and its precision (such as 95% confidence interval); any correlations and time effects estimated in the analysis.	N/A
	17b	For binary outcomes, presentation of both absolute and relative effect sizes is recommended.	N/A
Ancillary analyses	18	Results of any other analyses performed, including subgroup analyses and adjusted analyses, distinguishing pre-specified from exploratory.	N/A
Harms	19	Important harms or unintended effects in each treatment condition (for specific guidance see CONSORT for harms).	N/A
**Discussion**			
Limitations	20	Trial limitations, addressing sources of potential bias, imprecision, and, if relevant, multiplicity of analyses.	20

Generalisability	21	Generalisability (external validity, applicability) of the trial findings. Generalisability to clusters and/or individual participants (as relevant).	20
Interpretations	22	Interpretation consistent with results, balancing benefits and harms, and considering other relevant evidence.	N/A
**Other information**			
Registration	23	Registration number and name of trial registry.	21
Protocol	24	Where the full trial protocol can be accessed, if available.	21
Funding	25	Sources of funding and other support (such as supply of drugs), role of funders.	21
Research Ethics review	26	Whether the study was approved by a research ethics committee, with identification of the review committee(s). Justification for any waiver or modification of informed consent requirements.	21

This check list has been taken from table 3 in *BMJ* 2018;363:k1614, as a standalone document for readers to print out or fill in electronically

Supplementary material 5: Checklist 4 – Template for intervention description and replication (TIDieR) checklist

Item number	Item	Where located **	
		Primary paper (page or appendix number)	Other † (details)
	**BRIEF NAME**	Page 1	
**1.**	Provide the name or a phrase that describes the intervention.<br>Enhancing Palliative Care in ICU (EPIC)	______________	_____________
	**WHY**	Pages 6	
**2.**	Describe any rationale, theory, or goal of the elements essential to the intervention.<br>The rationale is to integrate palliative care (PC) into ICU workflows to reduce ICU length of stay (LOS), alleviate patient/family distress, and optimize resource use.	______________	_______________
	**WHAT**	Pages 10-11	
**3.**	Materials: Describe any physical or informational materials used in the intervention, including those provided to participants or used in intervention delivery or in training of intervention providers.<br>Provide information on where the materials can be accessed (e.g. online appendix, URL).<br>Materials include the 6-item trigger checklist, telemedicine tools for palliative consultations, and educational materials (e.g., e-modules, webinars).	______________<br><br>Pages 10-11	_______________
**4.**	Procedures: Describe each of the procedures, activities, and/or processes used in the intervention, including any enabling or support activities.<br>Procedures include: screening ICU patients using the 6-item checklist, conducting tele-PC consultations, and delivering blended-learning training to ICU staff.	______________	_______________
	**WHO PROVIDED**	Pages 11-12	
**5.**	For each category of intervention provider (e.g. psychologist, nursing assistant), describe their expertise, background and any specific training given.<br>Intervention providers (EPIC-trained specialists in palliative care) deliver tele-consultations and guide ICU staff. Training includes webinars, e-modules, and practice sessions.	______________	_______________
	**HOW**	Pages 11–12	
**6.**	Describe the modes of delivery (e.g. face-to-face or by some other mechanism, such as internet or	______________	_______________

	telephone) of the intervention and whether it was provided individually or in a group.<br>Delivered via telemedicine (audio-visual platforms) and blended-learning (asynchronous e-modules and live sessions). Interventions are individualized to patient/family needs.		
	**WHERE**	Pages 9	
**7.**	Describe the type(s) of location(s) where the intervention occurred, including any necessary infrastructure or relevant features.<br>Interventions occurre in ICUs across five European countries, utilizing secure telemedicine platforms to overcome geographic and infrastructure barriers.	______________	______________
	**WHEN and HOW MUCH**	Pages 10–11	
**8.**	Describe the number of times the intervention was delivered and over what period of time including the number of sessions, their schedule, and their duration, intensity or dose.<br>Tele-PC consultations are conducted as needed during ICU stays (average 7–9 days). Educational interventions include monthly online sessions and quarterly audits.	______________	______________
	**TAILORING**	Pages 11	
**9.**	If the intervention was planned to be personalised, titrated or adapted, then describe what, why, when, and how.<br>The intervention is personalized based on patient/family needs and ICU staff requirements, using structured workflows and "champion" feedback.	______________	______________
	**MODIFICATIONS**	N/A	
**10.**ǂ	If the intervention was modified during the course of the study, describe the changes (what, why, when, and how).<br>Quarterly audits monitored fidelity. Adjustments will be made based on feedback from ICU teams and champions to address specific site needs.	______________	______________
	**HOW WELL**	N/A	
**11.**	Planned: If intervention adherence or fidelity was assessed, describe how and by whom, and if any strategies were used to maintain or improve fidelity, describe them.<br>Intervention fidelity is monitored quarterly through audits, tele-consultation logs, and adherence to the trigger checklist, as outlined in the Fidelity Monitoring Plan.	______________	______________
**12.**ǂ	Actual: If intervention adherence or fidelity was assessed, describe the extent to which the intervention was delivered as planned.	N/A<br>____________	

	Adherence data is collected using tele-consultation reports and e-CRFs. Outcomes will be evaluated in the final analysis.		______________ _

** **Authors** - use N/A if an item is not applicable for the intervention being described. **Reviewers** – use '?' if information about the element is not reported/not sufficiently reported.

† If the information is not provided in the primary paper, give details of where this information is available. This may include locations such as a published protocol or other published papers (provide citation details) or a website (provide the URL).

ǂ If completing the TIDieR checklist for a protocol, these items are not relevant to the protocol and cannot be described until the study is complete.

* We strongly recommend using this checklist in conjunction with the TIDieR guide (see *BMJ* 2014;348:g1687) which contains an explanation and elaboration for each item.

* The focus of TIDieR is on reporting details of the intervention elements (and where relevant, comparison elements) of a study. Other elements and methodological features of studies are covered by other reporting statements and checklists and have not been duplicated as part of the TIDieR checklist. When a **randomised trial** is being reported, the TIDieR checklist should be used in conjunction with the CONSORT statement (see www.consort-statement.org) as an extension of **Item 5 of the CONSORT 2010 Statement.** When a **clinical trial protocol** is being reported, the TIDieR checklist should be used in conjunction with the SPIRIT statement as an extension of **Item 11 of the SPIRIT 2013 Statement** (see www.spirit-statement.org). For alternate study designs, TIDieR can be used in conjunction with the appropriate checklist for that study design (see www.equator-network.org).

Supplementary material 6: Methodological details 1 – Description of the telepalliative care consultation content

- **Which criteria from the EPIC checklist apply to this patient?**
- **Were there refractory symptoms before telepalliative care consultation which the ICU team found difficult to handle?**
  - ◯ Physical symptoms (e.g. pain, dyspnoea, patient burden from invasive measures, vomiting)
  - ◯ Psychological distress of the patient (e.g. lack of understanding, prognostic awareness, anxiety, fear)
  - ◯ Psychological distress of the family
  - ◯ Social problems (e.g. support of kids, financial, discharge location, social counselling
  - ◯ Spiritual care ◯ None

- According to the ICU physician: do **social problems** exist?
- Are **patient wishes and values** known before telepalliative care consultation?
- Are patient wishes and values **documented** before telepalliative care consultation?
- Does the patient and/or the patient family **understand prognosis**?
- Was this understanding **documented**?
- Was **Shared decision-making** documented before telepalliative care consultation?
- Was a **treatment limitation** documented before telepalliative care consultation?
- Type of limitation:
  - ◯ None/Full code ◯ Unknown
  - ◯ Withhold (explanation: do not escalate, do not dialyze, do not ...)
  - ◯ Withdraw
  - ◯ NACPR (explanation: do not attempt cardiopulmonary resuscitation)
  - ◯ Time-limited trial TLT (explanation: time limited trial of intensive care treatment)
- Present living will, healthcare proxy or legal guardian?
- During the telepalliative care consultation was an **interview with patient/relatives** performed by interventionists?
- Were **symptoms reviewed and possible interventions** discussed?
- Which symptoms?: Physical / Psychological / Social / Spiritual
- Was **patient / family understanding** assessed and its documentation recommended?
- Patient/family understanding: Illness/ Prognosis / Treatment options
- Were **patient-centred goals of care** assessed and their documentation recommended?
- If yes, this included one / more of the following:
  - ◯ Patients' values and priorities explored
  - ◯ Matching treatment alternatives discussed with patient/family
  - ◯ Goals of care documented
  - ◯ Treatment limitation established
  - ◯ Discharge plan formulated

Supplementary material 7: References supporting the selection of the secondary outcomes

In-hospital clinical outcome measures

1. Vincent JL, Moreno R, Takala J, et al. The SOFA (Sepsis-related Organ Failure Assessment) score. Results of a multicentre, prospective study. Intensive Care Med. 1996;22(7):707–710.
2. Ely EW, Inouye SK, Bernard GR, et al. Delirium in mechanically ventilated patients: validity and reliability of the Confusion Assessment Method for the ICU (CAM-ICU). Crit Care Med. 2001;29(7):1373–1380.
3. Fernandez-Bussy S, et al. Tracheostomy tube placement: early and late complications. J Bronchology Interv Pulmonol. 2017;24(2):202–210.
4. Christensen L, Jensen HI, Kristensen S, et al. Treatment limitations in intensive care units. Dan Med J. 2021;68(8):A03210235.
5. Yin YL, Sun MR, Zhang K, Chen YH, Zhang J, Zhang SK, et al. Status and risk factors in patients requiring unplanned intensive care unit readmission within 48 hours: a retrospective propensity-matched study. Risk Manag Healthc Policy. 2023;16:383–391.
6. European Centre for Disease Prevention and Control. Hospital discharges and length of stay statistics. Eurostat. Updated October 2024. https://ec.europa.eu/eurostat/statistics-explained/index.php/Hospital_discharges_and_length_of_stay_statistics. Accessed May 30, 2025.
7. Plotnikoff KM, Krewulak KD, Hernández L, et al. Patient discharge from intensive care: an updated scoping review to identify tools and practices to inform high-quality care. Crit Care. 2021;25:438.
8. Kaier K, Heister T, Wolff J, Wolkewitz M. Mechanical ventilation and the daily cost of ICU care. BMC Health Serv Res. 2020;20:267.
9. Kalzén H, von Bahr V, Palmer K, Frenckner B. Long term outcome after respiratory ECMO and length of ECMO treatment. Intensive Care Med Exp. 2015;3(Suppl 1):A153.
10. Chen WY, Cai LH, Zhang ZH, Tao LL, Wen YC, Li ZB, et al. The timing of continuous renal replacement therapy initiation in sepsis-associated acute kidney injury in the intensive care unit: the CRTSAKI Study protocol. BMJ Open. 2022;11(2):e040718.
11. Kjaergard LL, et al. Extracorporeal liver support in acute liver failure: a systematic review. Crit Care. 2017;21:7.
12. Rosenbaum ML, et al. Ventricular assist device support duration and outcomes: an analysis of national data. J Heart Lung Transplant. 2020;39(8):933–940.

In-hospital patient-centred and palliative care outcome measures

1. Porter LL, Simons KS, Corsten S et al. Changes in quality of life 1 year after intensive care: a multicenter prospective cohort of ICU survivors. Crit Care. 2024;28:255.

Patient follow up outcomes (3 month follow up)

1. Samuelson K. The Stressful Memory Assessment Checklist for the Intensive Care Unit (SMAC-ICU): Development and Testing. Healthcare. 2022;10(7):1321.
2. Adzrago D, Walker TJ, Williams F. Reliability and validity of the Patient Health Questionnaire-4 scale and its subscales of depression and anxiety among US adults. BMC Psychiatry. 2024;24:213.
3. Bergbom I, Karlsson V, Ringdal M. Developing and evaluating an instrument to measure Recovery After Intensive care: the RAIN instrument. BMC Nursing. 2018;17:5.
4. Prevedello D, Fiore M, Creteur J, Preiser JC. Intensive care units follow-up: a scoping review protocol. BMJ Open. 2020;10(11):e037725.
5. Laurent A, Fournier A, Lheureux F, et al. An international tool to measure perceived stressors in intensive care units: the PS-ICU scale. Ann Intensive Care. 2021;11:57.
6. Korsah EK, Schmollgruber S. Barriers and facilitators to end-of-life care in the adult intensive care unit: A scoping review. Int J Afr Nurs Sci. 2023;19:100636.
7. Ribet Buse E, Grunow JJ, Spies CD, Weiss B, Paul N. Health-related quality of life correlates with patient-reported and proxy-reported disability in critical illness survivors: a secondary analysis of the ERIC trial. Crit Care. 2025;29:158.
8. Effendy C, Yodang Y, Amalia S, Rochmawati E. Barriers and facilitators in the provision of palliative care in adult intensive care units: a scoping review. Acute Crit Care. 2022;37(4):516-526.
9. Walshe C, Dunleavy L, Preston N, et al. Understanding barriers and facilitators to palliative and end-of-life care research: a mixed-method study. BMC Palliat Care. 2024;23:159.
10. Jensen HI, Ammentorp J, Bjørnshave K, Møller Nielsen JF. Communication skills training in end-of-life care: A randomized controlled trial in critical care settings. Palliat Med. 2019;33(3):284-296.
11. Jensen HI, Lipczinsky A, Dahlgaard J, Langhoff H, Boye LK. Barriers and facilitators to integrating palliative care in intensive care: A mixed-methods study among ICU clinicians. Crit Care Med. 2021;49(5):e450-e459.
12. Jensen HI, Schou B, Nielsen JF, et al. Ethical challenges in palliative care decision-making in the ICU: Perspectives from healthcare providers. BMC Palliat Care. 2020;19(1):76.
13. Jensen HI, Stensballe J, Skriver M, Ammentorp J. Quality of end-of-life care in the ICU: Development of a standardized assessment tool. J Crit Care. 2022;67:109-117.
14. Jensen HI, Lauridsen SM, Dreyer P. Factors influencing decision-making regarding palliative care in the ICU: Insights from Danish intensive care clinicians. Acta Anaesthesiol Scand. 2023;67(7):945-954.

Relative follow up outcomes (3 month follow up)

1. Haave RO, Bakke HH, Schröder A. Family satisfaction in the intensive care unit: a cross-sectional study from Norway. BMC Emerg Med. 2021;21:20.
2. Jensen HI, Gerritsen RT, Koopmans M, et al. Satisfaction with quality of ICU care for patients and families: the euroQ2 project. Crit Care. 2017;21:239.
3. Kerckhoffs MC, et al. Framework to support the process of decision-making on life-sustaining treatments in the ICU. Crit Care Med. 2020;48(5):645-653.
4. Rensen A, van Mol MM, Menheere I, et al. Quality of care in the ICU from the perspective of patient's relatives: development and psychometric evaluation of the consumer quality index 'R-ICU'. BMC Health Serv Res. 2017;17:77.
5. Frivold G, Slettebø Å, Heyland DK, et al. Family members' satisfaction with care and decision-making in intensive care units and post-stay follow-up needs—a cross-sectional survey study. Nurs Open. 2017;4(2):97.
6. Clark K, Milner KA, Beck M, Mason V. Measuring family satisfaction with care delivered in the ICU. Crit Care Nurse. 2016;36(6):e8-e14.
7. Kursumovic E, Bilinska J, Molokhia A. Family satisfaction in the ICU: enhancing patient experience. Crit Care. 2013;17:P544.
8. Nakahara R, Weibel L, Zollinger A, Hofer C. Quality of life after prolonged ICU stay: preliminary results of a prospective survey in critically ill patients. Crit Care. 2008;12:P505.
9. Stajduhar K, Sawatzky R, Cohen SR, et al. Bereaved family members' perceptions of the quality of end-of-life care across four types of inpatient care settings. BMC Palliat Care. 2017;16:59.
10. Minton ME, Isaacson MJ, Da Rosa P. Psychometric analysis of the Comfort with Communication in Palliative and End-of-Life Care (C-COPE) instrument. Palliat Med. 2020;34(5):678-689.
11. Effendy C, Yodang Y, Amalia S, Rochmawati E. Barriers and facilitators in the provision of palliative care in adult intensive care units: a scoping review. Acute Crit Care. 2022;37(4):516-526.
12. Walshe C, Dunleavy L, Preston N, et al. Understanding barriers and facilitators to palliative and end-of-life care research: a mixed-method study. BMC Palliat Care. 2024;23:159.

Resource utilization outcomes

1. Rothen HU, Stricker K, Einfalt J, et al. Variability in outcome and resource use in intensive care units. Intensive Care Med. 2007;33(8):1329-1336.
2. Kochanek M, Berek M, Janssens U, Kitz V, Wilkens FM. Überversorgung und Nachhaltigkeit in der Intensiv- und Notfallmedizin [Intensive care and emergency medicine overuse and sustainability]. Med Klin Intensivmed Notfmed. 2025;120(1):22-29.

Health economic outcomes

Costs

1. Seidel J, Whiting PC, Edbrooke DL. The costs of intensive care, *Continuing Education in Anaesthesia Critical Care & Pain. 2006;6(4):160-163.*
2. Tan SS, Bakker J, Hoogendoorn ME, et al. Direct cost analysis of intensive care unit stay in four European countries: applying a standardized costing methodology. Value Health. 2012;15(1):81-86.
3. Bruyneel A, Larcin L, Martins D, Van Den Bulcke J, Leclercq P, Pirson M. Cost comparisons and factors related to cost per stay in intensive care units in Belgium. BMC Health Serv Res. 2023;23(1):986.
4. Bruyneel A, Larcin L, Tack J, Van Den Bulcke J, Pirson M. Association between nursing cost and patient outcomes in intensive care units: A retrospective cohort study of Belgian hospitals. Intensive and Critical Care Nursing 2022; 73:103296.
5. Fialová, Š. Costing in conditions of Czech hospitals. 2013. https://aak.slu.cz/pdfs/aak/2013/04/04.pdf
6. Tichopad A, Roberts C, Gembula I, et al. Clinical and economic burden of community-acquired pneumonia among adults in the Czech Republic, Hungary, Poland and Slovakia. PLoS One. 2013;8(8):e71375.
7. Chacko B, Ramakrishnan N, Peter JV. Approach to Intensive Care Costing and Provision of Cost-effective Care. Indian J Crit Care Med. 2023;27(12):876-887.
8. Mastrogianni M, Galanis P, Kaitelidou D, Konstantinou E, Fildissis G, Katsoulas T. Factors affecting adult intensive care units costs by using the bottom-up and top-down costing methodology in OECD countries: A systematic review. Intensive Crit Care Nurs. 2021;66:103080.

Quality of life

1. Lau VI, Johnson JA, Bagshaw SM, Rewa OG, Basmaji J, Lewis KA, et al. Health-related quality-of-life and health-utility reporting in critical care. World J Crit Care Med 2022;11(4): 236-245.
2. Lawson A, Tan AC, Naylor J, Harris IA. Is retrospective assessment of health-related quality of life valid?. BMC Musculoskelet Disord. 2020;21(1):415.

Clinician-related outcome measures

1. Schaufeli WB, De Witte H, Desart S. Manual Burnout Assessment Tool (BAT) Version 2.0. Leuven, Belgium: KU Leuven; 2020.
2. Piers RD, Azoulay E, Ricou B, et al. Perceptions of appropriateness of care among European and Israeli intensive care unit nurses and physicians. JAMA. 2011;305(6):597–603.
3. Peng M, Guan Q, Zhu X. Moral distress, attitude toward death, and palliative care core competencies among ICU nurses: A cross-sectional study. BMC Palliat Care. 2025;24:16.

4. The EOLE Study Group. End-of-life in the critically ill patient: evaluation of experience of end-of-life by caregivers (EOLE study). Ann Intensive Care. 2021;11:162.
5. Van den Bulcke B, Piers R, Jensen HI, et al. Ethical decision-making climate in the ICU: theoretical framework and validation of a self-assessment tool. BMJ Qual Saf. 2018;27(10):781–787.
6. Agency for Healthcare Research and Quality. Hospital Survey on Patient Safety Culture Version 2.0. Rockville, MD: AHRQ; 2021.
7. Stollings JL, Devlin JW, Puntillo KA, et al. Implementing the ABCDEF Bundle: Top 8 Questions Asked During the ICU Liberation ABCDEF Bundle Improvement Collaborative. Crit Care Nurse. 2019;39(1):36–44.
8. Levy M, Curtis JR, Luce J, Nelson JD, Clarke EB. Measuring the Quality of Palliative Care in the Intensive Care Unit. Seattle, WA: University of Washington; 2011.
9. National and Kapodistrian University of Athens. Survey on Palliative Care and Law Perception (PCLAW). ClinicalTrials.gov identifier: NCT06245525; 2023.
10. Effendy C, Yodang Y, Amalia S, Rochmawati E. Barriers and facilitators in the provision of palliative care in adult intensive care units: a scoping review. Acute Crit Care. 2022;37(4):516–526.
11. Walshe C, Dunleavy L, Preston N, et al. Understanding barriers and facilitators to palliative and end-of-life care research: a mixed-method study. BMC Palliat Care. 2024;23:159.
12. Schwartzkopf D, Westermann I, Skupin H, et al. End-of-Life Decision-making and Staff Stress Questionnaire (EIDECS). PsycTESTS. 2015. doi:10.1037/t80003-000
13. Mentzelopoulos SD, Chen S, Nates JL, et al; on behalf of the End-of-Life Practice Score Study Group. Derivation and performance of an end-of-life practice score aimed at interpreting worldwide treatment-limiting decisions in the critically ill. Crit Care. 2022;26:106.

Supplementary material 8: Figure 2 – Detailed outcome overview

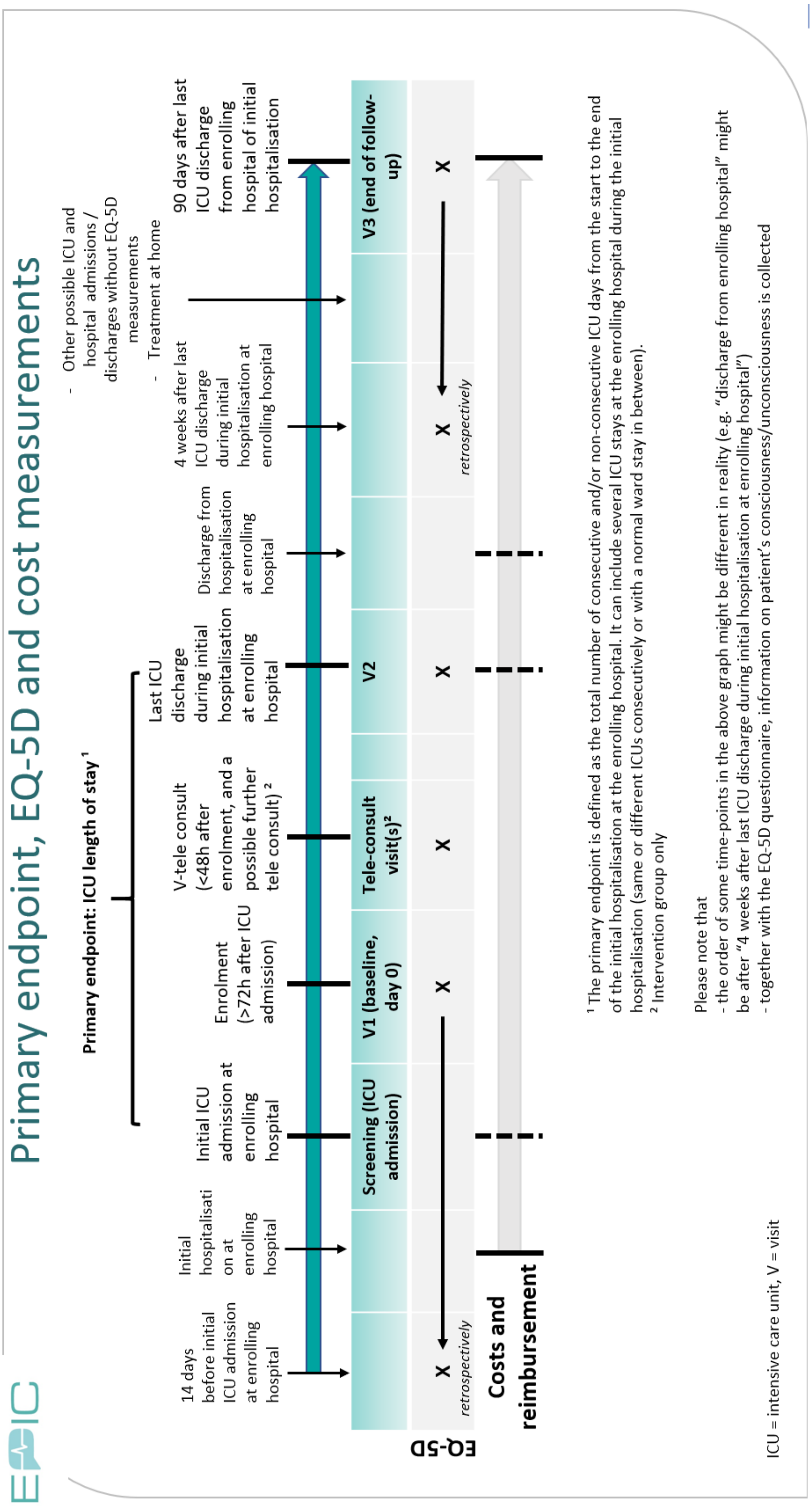

Supplementary material 9: Methodological details 2 – Data management, pseudonymization, data transfer and archiving

Data collection and management

Three types of data will be collected in the EPIC trial from data subjects (patients, relatives, employees): clinical data, cost data and survey data. Before the study start, a data management plan was developed.

All clinical data collected during the study are recorded and stored in the eCRF using the secuTrial® database (interactive Systems GmbH, Berlin, Germany) hosted by Charité - Universitätsmedizin Berlin and directly analysed from this database. Follow-up data are collected initially via paper CRFs and questionnaires and then entered into the eCRF. Study staff at each centre load up clinical and cost data (per patient and per hospital) in the eCRF. An authorized member at each study centre signs all eCRFs electronically. This confirms that all data in the eCRF are correct and have not been changed. If a value on the eCRF is changed later on, the electronic signature is set back automatically and the corresponding page has to be signed again. This ensures that changes on the eCRF will be dated and signed as well. All entries and data changes are tracked automatically including date, time and person who entered/changed information (audit trail). Major correction or major missing data have to be explained. However, the investigator has final responsibility for the accuracy and authenticity of all clinical and economic data entered in the eCRF. All information required by the protocol, and collected during the clinical study, must be verified by source data (e.g., patient records, hospital controlling). The investigator and trial staff are responsible for ensuring adequate documentation of source data. Cost data of study participants will be processed by centres in pseudonymized excel-sheets and will be transmitted in encrypted form via secuTrial® or 7-Zip by email to the University of Basel or can be uploaded into the secuTrial® database. For the finalization of the Health Economic Analysis Plan and for the preparatory programming of the analyses, partial data extracts after 12, 24 and 30 months with pseudonymized patient-related data from the study database (secuTrial®) will be aggregated by the study team of the Charité Department of Anesthesiology and Intensive Care Medicine (CCM | CVK) and provided from data managers of the Clinical Trials Office (CTO) in encrypted form to the University of Basel. All relevant trial documents (Trial Master File-, the electronically stored data, including the eCRFs, the datasets and the final report) will be stored for 10 years after the study's completion. All study data and applications are physically stored in dedicated data centres on servers within Charité and University of Basel. Data collected through secuTrial® will be stored in an Oracle database (Windows Server – secuTrial® (6.5.1.7). Data collected through REDCap® will be stored in a MySQL database physically located on Charité server. At the coordinating sites, all completed study related documents (e.g., coordinating site files, follow up questionnaires (paper-CRF) and initiation visit, staff signature lists) will be stored for 10 years after the study's completion. With this time span all local rules and legal requirements regarding archiving at all sites will be addressed, including archiving telemedical visits. At the satellite sites, all completed study related documents (e.g., investigator site files, signed written consent forms, all paper CRFs, initiation visit, staff signature lists) will be stored for 10 years after the study's

completion. With this time span all local rules and legal requirements regarding archiving at all sites will be addressed.

Team surveys (ICU team, palliative care team) are documented in a REDCap® (REDCap Consortium, Vanderbilt University Medical Center, US) database hosted by Charité.

Pseudonymization

The pseudonyms for the trial patients and relatives are generated using the validated electronic eCRF. The pseudonymization list is stored in the electronic secuTrial® system at each research centre. The pseudonymization key (allocation list) can only be accessed by the principal investigator, authorized trial personnel, and authorized employees of the coordinating centres. It will be destroyed 10 years after the end of the trial. All records that can be used to identify the patient are subject to medical confidentiality and will be treated in strict confidence.

Data management plan

All codebooks from secuTrial® and REDCap® will be provided separately in a data repository (Zenodo®), along with the anonymized datasets. The data management procedures are described in detail in the Data Management Plan (DMP), which will be regularly updated throughout the project period.

Supplementary material 10: Figure 3 – Decision making structure

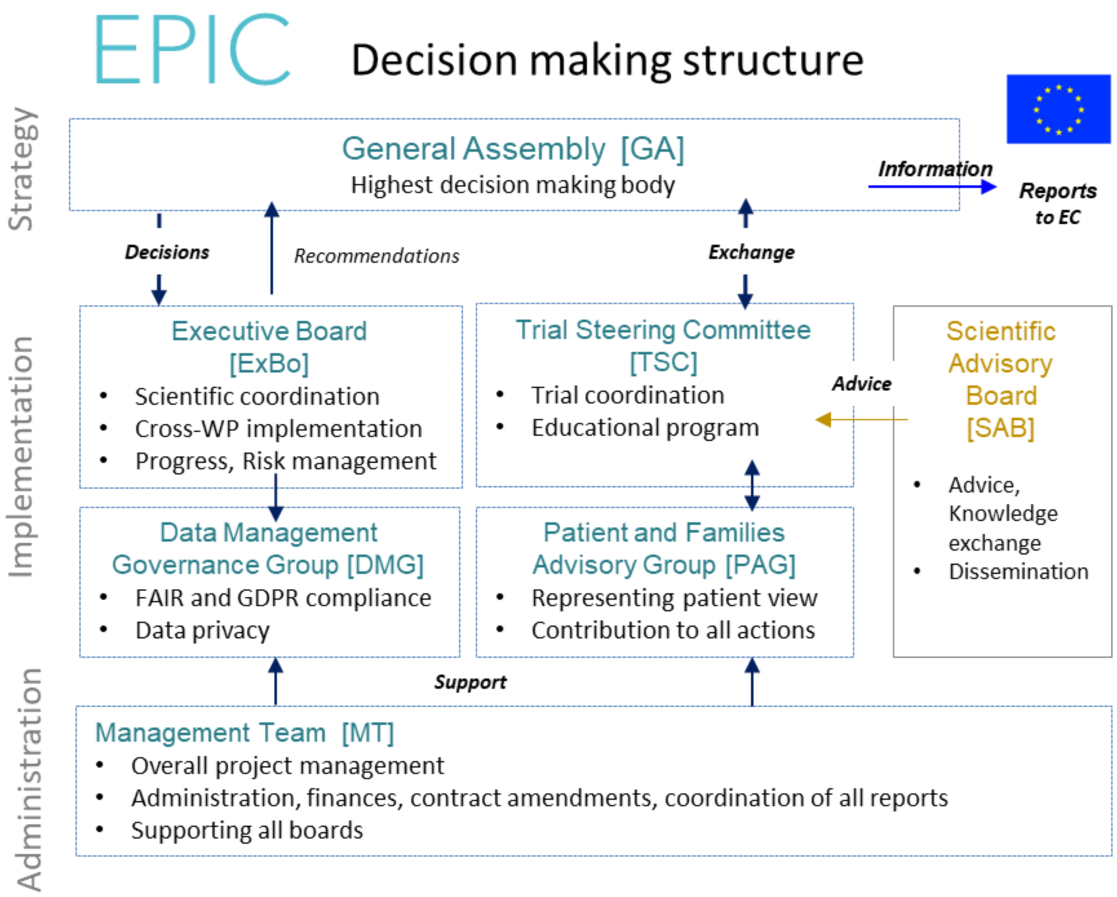

Supplement: online supplemental file 1 [file bmjopen-16-2-s001.pdf]
