# Supplementary material for: Enhancing palliative care in intensive care units: protocol of EPIC, a controlled, cluster-randomised, non-blinded stepped-wedge design trial with crossover phase
Source: BMJ Open. 2026 Feb 17;16(2):e108168. doi: 10.1136/bmjopen-2025-108168 (PMC12918684; doi:10.1136/bmjopen-2025-108168)
Supplement: Supplementary file 2 [file bmjopen-2025-108168.reviewer_comments.pdf]

PEER REVIEW HISTORY

BMJ Open publishes all reviews undertaken for accepted manuscripts. Reviewers are asked to complete a checklist review form and are provided with free text boxes to elaborate on their assessment. These free text comments are reproduced below.

ARTICLE DETAILS

Title (Provisional)

Enhancing palliative care in intensive care units: protocol of EPIC, a controlled, cluster-randomized, non-blinded stepped-wedge design trial with crossover phase

Authors

Mentzelopoulos, Spyros D; Hartog, Christiane S; Tenge, Dr. med. Theresa; Schwenkglenks, Matthias; Piper, Sophie K. K.; Barbier, Michaela Carla; Rusinova, Katerina; Neukirchen, Martin; Schüürhuis, Stephen; Jensen, Hanne Irene; van Heerden, Peter Vernon; Dutzmann, Jochen; Drescher, Dominique; Markéta, Zvara; Metaxa, Victoria; Nachshon, Akiva; De Robertis, Edoardo; Spies, Claudia; Edel, Andreas

---

VERSION 1 - REVIEW

**Reviewer**	**1**
**Name**	**Petrillo, Laura**
**Affiliation**	**Massachusetts General Hospital**
**Date**	**15-Nov-2025**
**COI**	**None**

This is a protocol paper for a controlled, cluster-randomized, non-blinded stepped-wedge design trial comparing an ICU tele-palliative care intervention to usual care. This is a comprehensive program that involves training for ICU clinicians about palliative care and implementation of bedside tools (a checklist) to identify patients eligible for palliative care, and delivery of palliative care by telehealth.

Major points:

- The main outcome of the study is hospital length of stay. However, the inclusion criteria includes “Need for specialist palliative care,” suggesting that the only patients “enrolled” in the study are those in the intervention period who meet criteria for palliative care. For patients in the observation period, are they providing informed consent to participate in the study? (it seems like no). Are all patients who are hospitalized during the observation period included in the control group, or only those who meet the criteria of needing palliative care? The way it is written, it seems like only patients who meet criteria for palliative care during the intervention period are providing consent and included in the intervention period LOS calculation, but this is not going to be comparable to the observation period, since it is only a subset – a sicker subset (though noted that patients expected to die in <24 hours are excluded). Is this correct? Please justify or clarify who is included in the LOS comparison.

- Regarding the above, if patients in the observation period are not providing informed consent, are they asked permission for accessing their medical record? Are they being asked to complete patient-reported outcomes? If they are not, what are the intervention patients' outcomes being compared to?

Minor points:

- Inclusion criteria: please provide justification for excluding patients with cancer

---

VERSION 1 - AUTHOR RESPONSE

Reviewer: 1
Dr. Laura Petrillo, Massachusetts General Hospital
Comments to the Author:
This is a protocol paper for a controlled, cluster-randomized, non-blinded stepped-wedge design trial comparing an ICU tele-palliative care intervention to usual care. This is a comprehensive program that involves training for ICU clinicians about palliative care and implementation of bedside tools (a checklist) to identify patients eligible for palliative care, and delivery of palliative care by telehealth.
Response: Thank you for reviewing our study protocol and for your valuable feedback.

Major points:
- The main outcome of the study is hospital length of stay.
Response: We respectfully clarify that the primary outcome of the study is ICU length of stay, not hospital length of stay. This is clearly stated in the study protocol:

Objectives and Hypotheses (page 7)
"The primary hypothesis is that the complex intervention will reduce the average ICU LOS by at least two days compared to standard care before."
Outcome Measures (page 12)
"Primary outcome
The primary outcome is the ICU LOS in days, operationalized as the total number of consecutive and/or non-consecutive ICU days from the start to the end of an index hospitalization at a study-participating hospital; any transfer to another hospital for treatment continuation signifies termination of the index hospitalization, and therefore, any subsequent ICU admission cannot contribute to the measured ICU LOS. Regarding index hospitalization, whenever ICU discharge is followed by ICU readmission on the same day, ICU LOS is determined by taking into account the aforementioned day only once (rather than twice)."

However, the inclusion criteria includes “Need for specialist palliative care,” suggesting that the only patients “enrolled” in the study are those in the intervention period who meet criteria for palliative care. For patients in the observation period, are they providing informed consent to participate in the study? (it seems like no).

Response: Thank you for this comment. We have already clearly addressed in the “Inclusion criteria” (page 10) that *"ICU patients with decisional capacity, who can give informed consent for study participation, and incapacitated patients with an available, legally recognized, substitute decisionmaker able to provide informed consent on their behalf"* are eligible.
To further clarify, we have revised "Eligibility Criteria" to read as follows:
"Identical enrolment criteria apply for both the observation and intervention phase; therefore, throughout the study period, all consecutive patients who are admitted to a participating ICU will be routinely screened for the following criteria."

Are all patients who are hospitalized during the observation period included in the control group, or only those who meet the criteria of needing palliative care? The way it is written, it seems like only patients who meet criteria for palliative care during the intervention period are providing consent and included in the intervention period LOS calculation, but this is not going to be comparable to the observation period, since it is only a subset – a sicker subset (though noted that patients expected to die in <24 hours are excluded). Is this correct? Please justify or clarify who is included in the LOS comparison.
Response: We apologize for any lack of clarity. In both the observation (control) and intervention periods, identical enrolment criteria are applied, and all consecutive ICU patients who meet all eligibility criteria are enrolled, regardless of study phase. Thus, the LOS comparison is based on the same patient population in both periods and is not restricted to a sicker subset during the intervention phase.
To clarify this explicitly, we have revised the first two sentences of the “Eligibility Criteria” subsection to read (page 10): *“Identical enrolment criteria apply for both the observation and intervention phases; therefore, throughout the study period, all consecutive patients admitted to a participating ICU are routinely screened for the same eligibility criteria.”*
Accordingly, in all participating centres, patients in both the control and intervention groups must fulfil the same criteria, minimizing the risk of comparability bias between study phases. While the need for specialized palliative care may arise in any eligible ICU patient during either phase, the distinction between groups lies in the care

delivered: control patients receive standard local care (which may not include organized palliative care), whereas intervention patients receive structured, teleconsultation-supported palliative care aimed at addressing existing gaps in ICU palliative care practice.

- Regarding the above, if patients in the observation period are not providing informed consent, are they asked permission for accessing their medical record? Are they being asked to complete patient-reported outcomes? If they are not, what are the intervention patients' outcomes being compared to?

Response: Patients enrolled during the observation period also provide written informed consent, identical to patients enrolled during the intervention period. Consent covers access to medical records as well as the collection of patient-reported outcomes (e.g., EQ-5D-5L). Therefore, outcomes assessed in the intervention period are directly compared with outcomes collected from consented patients meeting the same eligibility criteria during the observation period.

Consent (page 19)

"Individual written informed consent including consent to data collection will be obtained from all eligible patients, or their legal guardians, or their next-of-kin by the study personnel. The next-of-kin consent will be considered as valid, provided that it is explicitly recognized by local law."

Minor points:

- Inclusion criteria: please provide justification for excluding patients with cancer

Response: Thank you for raising this important aspect. Please find our reasons for not including patients with cancer as follows:

1. Distinct illness trajectory and prognosis

   Cancer patients admitted for cancer-related complications often have different survival trajectories and treatment intent (disease-directed vs purely comfort-focused) compared with patients who develop critical illness from non-malignant causes. That heterogeneity can change both the baseline need for palliative care and the expected responsiveness to an ICU focused PC intervention [1].

2. High baseline palliative involvement and specialized pathways

   Many oncology services already integrate palliative care earlier and more systematically, and cancer patients may have preexisting PC relationships, advance care planning, or oncology-driven goals-of-care processes. Including

them risks confounding because the intervention's incremental effect over usual care would be smaller or variable [1].

3. Different decision-making dynamics and family expectations
   Goals of care discussions, prognostic uncertainty, and decisions about limiting life-sustaining therapy often follow different patterns in advanced cancer, influenced by oncologic prognosis, ongoing systemic therapy options, and prior discussions—factors that can interact with our intervention in unpredictable ways [2].
4. Risk of effect dilution and loss of internal validity
   If cancer-primary patients have systematically shorter or longer ICU LOS, different rates of treatment limitation, or different symptom profiles, they can dilute the measurable impact on our primary endpoint (ICU LOS) and secondary outcomes, making it harder to detect a true effect in the target population of non-cancer critical illness [2].
5. Heterogeneous interventions and resource needs
   Palliative needs in cancer (e.g., complex symptom control for tumor-related pain, oncologic emergencies) may require oncology specific expertise that our standardized telemedicine/ICU-focused PC model does not provide, reducing fidelity and interpretability of results [2].
6. Ethical and pragmatic considerations
   Trials should avoid enrolling groups for whom the intervention is already standard practice or where withholding the intervention would be ethically fraught. If many cancer patients already receive specialist PC, randomizing them into a stepped wedge implementation could raise ethical and logistical issues [1] [3].
7. Recent literature [1] [4] [5] strongly implies that the most significant evidence gap exists in the area of palliative care for critically ill patients without cancer. This further supports the decision to exclude critically ill cancer patients from the study.

References

[1] Schellongowski P, et al. Critically ill patients with cancer: chances and limitations of intensive care medicine-a narrative review. ESMO Open. 2016;1(5):e000018.

[2] Quinn KL, et al. Rethinking Palliative Interventions in Critical Care-When More Is Not Better. JAMA Intern Med. 2025;185(2):184-185

[3] Salins N, et al. Palliative and end-of-life care practices for critically ill patients and their families in a peri-intensive care setting: A protocol for an umbrella review. Palliat Support Care. 2024;22(5):1418-1425.
[4] Dumas G, Pastores SM, Munshi L. Five new realities in critical care for patients with cancer. Intensive Care Med. 2023;49:345-348.
[5] Quinn KL, Shurrab M, Gitau K, Kavalieratos D, Isenberg SR, Stall NM, Stukel TA, Goldman R, Horn D, Cram P, Detsky AS, Bell CM. Association of Receipt of Palliative Care Interventions With Health Care Use, Quality of Life, and Symptom Burden Among Adults With Chronic Noncancer Illness: A Systematic Review and Meta-analysis. JAMA. 2020;324:1439-1450.

Based on the above-provided arguments, we have revised the eligibility criterion, which now reads as follows:

"The leading cause of critical illness is not cancer; such patients represent a clinically distinct group with different prognosis, pre-existing palliative care involvement, and oncology specific care pathways that would likely confound the trial's effect on ICU length of stay and other outcomes [26] [27]. Moreover, the most significant evidence gap in the literature on PC for critically ill patients concerns non-cancer populations

[26] [27] [28], providing a strong rationale for the inclusion criterion and the design of the present trial."

References

[26] Schellongowski P, et al. Critically ill patients with cancer: chances and limitations of intensive care medicine-a narrative review. ESMO Open. 2016;1(5):e000018.
[27] Dumas G, Pastores SM, Munshi L. Five new realities in critical care for patients with cancer. Intensive Care Med. 2023;49:345-348

Accordingly, we added references 26 and 27 to the revised paper's reference list.

[28] Quinn KL, Shurrab M, Gitau K, Kavalieratos D, Isenberg SR, Stall NM, Stukel TA, Goldman R, Horn D, Cram P, Detsky AS, Bell CM. Association of Receipt of Palliative Care Interventions With Health Care Use, Quality of Life, and Symptom Burden Among Adults With Chronic Noncancer Illness: A Systematic Review and Meta-analysis. JAMA. 2020;324:1439-1450.
